# Sigma-1 and dopamine D2/D3 receptor occupancy of pridopidine in healthy volunteers and patients with Huntington disease: a [^18^F] fluspidine and [^18^F] fallypride PET study

**DOI:** 10.1007/s00259-020-05030-3

**Published:** 2020-09-29

**Authors:** Igor D. Grachev, Philipp M. Meyer, Georg A. Becker, Marcus Bronzel, Doug Marsteller, Gina Pastino, Ole Voges, Laura Rabinovich, Helena Knebel, Franziska Zientek, Michael Rullmann, Bernhard Sattler, Marianne Patt, Thilo Gerhards, Maria Strauss, Andreas Kluge, Peter Brust, Juha-Matti Savola, Mark F. Gordon, Michal Geva, Swen Hesse, Henryk Barthel, Michael R. Hayden, Osama Sabri

**Affiliations:** 1 Teva Branded Pharmaceutical Products R&D, Inc, Malvern, PA 19355 USA; 2 Guide Pharmaceutical Consulting, LLC, Millstone, NJ 08535 USA; 3 Department of Nuclear Medicine, University of Leipzig Medical Center, Leipzig, Germany; 4 ABX-CRO Advanced Pharmaceutical Services Forschungsgesellschaft mbH, Dresden, Germany; 5 Teva Branded Pharmaceutical Products R&D, Inc, Frazer, PA 19355 USA; 6 Department of Psychiatry and Psychotherapy, University of Leipzig Medical Center, Leipzig, Germany; 7 Helmholtz-Zentrum Dresden-Rossendorf, Institute of Radiopharmaceutical Cancer Research, Research Site Leipzig, Leipzig, Germany; 8 Prilenia Therapeutics Development Ltd., Herzliya, Israel

**Keywords:** [^18^F]fluspidine, PET, Pridopidine, Sigma-1 receptor occupancy, Dopamine D2/D3 receptor occupancy, Huntington disease

## Abstract

**Purpose:**

Pridopidine is an investigational drug for Huntington disease (HD). Pridopidine was originally thought to act as a dopamine stabilizer. However, pridopidine shows highest affinity to the sigma-1 receptor (S1R) and enhances neuroprotection via the S1R in preclinical studies. Using [^18^F] fluspidine and [^18^F] fallypride PET, the purpose of this study was to assess in vivo target engagement/receptor occupancy of pridopidine to the S1R and dopamine D2/D3 receptor (D2/D3R) at clinical relevant doses in healthy volunteers (HVs) and as proof-of-concept in a small number of patients with HD.

**Methods:**

Using [^18^F] fluspidine PET (300 MBq, 0–90 min), 11 male HVs (pridopidine 0.5 to 90 mg; six dose groups) and three male patients with HD (pridopidine 90 mg) were investigated twice, without and 2 h after single dose of pridopidine. Using [^18^F] fallypride PET (200 MBq, 0–210 min), four male HVs were studied without and 2 h following pridopidine administration (90 mg). Receptor occupancy was analyzed by the Lassen plot.

**Results:**

S1R occupancy as function of pridopidine dose (or plasma concentration) in HVs could be described by a three-parameter Hill equation with a Hill coefficient larger than one. A high degree of S1R occupancy (87% to 91%) was found throughout the brain at pridopidine doses ranging from 22.5 to 90 mg. S1R occupancy was 43% at 1 mg pridopidine. In contrast, at 90 mg pridopidine, the D2/D3R occupancy was only minimal (~ 3%).

**Conclusions:**

Our PET findings indicate that at clinically relevant single dose of 90 mg, pridopidine acts as a selective S1R ligand showing near to complete S1R occupancy with negligible occupancy of the D2/D3R. The dose S1R occupancy relationship suggests cooperative binding of pridopidine to the S1R. Our findings provide significant clarification about pridopidine’s mechanism of action and support further use of the 45-mg twice-daily dose to achieve full and selective targeting of the S1R in future clinical trials of neurodegenerative disorders.

Clinical Trials.gov Identifier: NCT03019289 January 12, 2017; EUDRA-CT-Nr. 2016-001757-41.

**Electronic supplementary material:**

The online version of this article (10.1007/s00259-020-05030-3) contains supplementary material, which is available to authorized users.

## Introduction

Pridopidine is an investigational drug under clinical development for the therapy of Huntington disease (HD) and amyotrophic lateral sclerosis (ALS). HD is a devastating neurodegenerative disease (NDD) with an autosomal-dominant inheritance. HD is clinically characterized by motor, psychiatric, and cognitive dysfunction. The causative genetic mutation is the expansion of the cytosine-adenine-guanine (CAG) trinucleotide repeat in the Huntingtin gene (*HTT*). Striatal and cortical neurons are particularly damaged and degenerate early and progressively in HD [1]. ALS is a terminating NDD associated with death of motor neurons leading to muscle atrophy, paralysis, and respiratory collapse within a mean of 3 to 5 years from symptom onset [2]. Up to now, there are no disease-modifying drugs for HD and ALS and the available treatment options are of limited efficacy.

Pridopidine has been originally thought to act as a dopamine stabilizer by modulating dopamine-dependent behaviors and acting as a low affinity dopamine D2 receptor (D2R) ligand [3, 4]. According to preclinical investigations, pridopidine was suggested to normalize motor function by either inhibiting dopamine-induced hyperlocomotion or enhancing the low baseline locomotor activity in habituated animals, without affecting normal locomotor activity [4, 5]. However, recent in vitro and in vivo animal studies revealed that pridopidine exerts highest affinity towards the sigma-1 receptor (S1R), showing ~ 30-fold higher affinity compared with the dopamine D3 receptor (D3R) and ~ 100-fold higher affinity compared with the D2R [6–8], indicating that pridopidine is working predominantly through the S1R.

The S1R is a chaperone protein located at the endoplasmic reticulum (ER)-mitochondrion interface and plays an important role for numerous physiological functions by modulating ER-nucleus cross talk and ER-mitochondrion signaling [9]. Upon ligand-activation, S1R promotes diverse cellular processes, including calcium and ion channel signaling, ER stress response, and mitochondrial function [9, 10]. These cellular pathways are commonly impaired in many NDD, including HD [1, 10]. Genetic findings show that loss of function mutations in the S1R are associated with juvenile ALS and distal hereditary motor neuropathies underpinning the role of S1R in the pathophysiology of NDDs [11, 12]. Importantly, S1R activation, e.g., by pridopidine, enhances neuroprotective effects in preclinical models of neurodegeneration, including HD and ALS, acting to stimulate brain repair and plasticity. Pridopidine augments brain-derived neurotrophic growth factor (BDNF) secretion and rescues dendritic spine loss and restores the aberrant calcium signaling via the S1R shown in experimental HD, Parkinson disease (PD), and ALS [13–17].

The PRIDE-HD was an exploratory phase 2 trial evaluating pridopidine at doses between 45 and 112.5 mg bidaily (bid) in HD patients [18]. Pridopidine 45 mg bid demonstrates significantly less decline from baseline in total functional capacity (TFC), compared with the placebo group at week 52. TFC is a validated clinical scale in HD used by clinicians to assess disease stage and monitor decline of functional capacity. In PRIDE-HD, the most pronounced and significant effect is observed with the dose of 45 mg bid (18,19).

In vivo target engagement/receptor occupancy of pridopidine, i.e., its binding to the S1R and the D2/D3R in the human brain is unknown. To clarify pridopidine’s mechanism of action, we used PET imaging to assess the receptor occupancy of pridopidine at previously used clinical doses. (S)-(-)-[^18^F] Fluspidine (termed here [^18^F] Fluspidine for simplicity) proved to be a selective and suitable radioligand for neuroimaging of S1R availability in preclinical PET studies and a recent first-in-human PET investigation [19–23]. (S)-(-)-[^18^F] Fluspidine shows a more favorable metabolic profile compared with its (R)-(-)-enantiomer, and its binding to the S1R is reversible, whereas binding of the (R)-(-)-enantiomer is irreversible [20]. [^18^F] fallypride is a well-characterized high-affinity, non-selective D2/D3R radioligand with a preference for the D2R [24]. [^18^F] fallypride has frequently been used in PET studies for the quantification of the D2/D3R in the brain and shows high specific binding [24, 25].

The primary objective of this PET study was to investigate the S1R occupancy of pridopidine in HVs and as proof-of-concept in a small number of HD patients using [^18^F] fluspidine PET. Another primary objective was to determine the relationship between pridopidine dose/plasma concentration and S1R occupancy in HVs. Secondary/exploratory objectives were to analyze the D2/D3R occupancy of pridopidine using [^18^F] fallypride PET, the pharmacokinetics and safety of pridopidine, and the test-retest variability of [^18^F] fluspidine PET.

## Materials and methods

This was a single-dose, open-label, adaptive design PET study to quantify the S1R and the D2/D3R occupancy of pridopidine in HVs and in a small number of patients with HD (Clinical Trials.gov Identifier: NCT03019289; EUDRA-CT-Nr. 2016-001757-41). This study was approved by the local ethics committee, the Federal Institute for Drugs and Medical Devices and the German Federal Office for Radiation Protection, and was performed according to the World Medical Association Declaration of Helsinki. This PET study was carried out at the Department of Nuclear Medicine, University Hospital of Leipzig, Germany. Written informed consent was obtained from all study participants.

### Subjects

Fifty-two male HVs and patients with HD were recruited according to specific criteria and extensively screened. Twenty HVs and three patients with HD were enrolled. Seventeen HVs (age 27.6 ± 2.7 years) and three patients with HD (age 43.3 ± 13.3 years) completed the study. Since we do not expect age-related effects on the receptor occupancy, in contrast to very possible, age-related effects on receptor density, HVs and HD patients were not matched for age. To reduce known variability of pridopidine plasma levels, poor metabolizers at the cytochrome P450 2D6 (*CYP2D6*) genotype were not enrolled. Patients with HD were clinically characterized at baseline using the UHDRS-Total Motor Score (TMS) and the UHDRS-Total Functional Capacity (TFC; Supplementary Materials and Methods; CONSORT-diagram, Supplementary Fig. S1) [26].

### Study design

This PET study consists of three PET substudies: the [^18^F] fluspidine substudy (HVs, *n* = 11, HD, *n* = 3), the [^18^F] fallypride substudy (HVs, *n* = 4), and the test-retest [^18^F] fluspidine substudy (HVs, *n* = 2). All subjects were assigned to only one PET substudy. Subjects in the [^18^F] fluspidine or [^18^F] fallypride substudies were investigated twice within 4 weeks at the same time point of the day. The first PET imaging was carried out at baseline, without prior pridopidine treatment (PET1) and the second imaging was performed 2 h after application of pridopidine (PET2). PET imaging started 2 h after pridopidine administration to correlate with the expected pridopidine plasma time to reach maximum (peak) concentration (*t*_max_) [27].

In the [^18^F] fluspidine substudy, the HVs (*n* = 11) were assigned to different cohorts according to the different doses of pridopidine. In HVs, an adaptive design was employed to determine the pridopidine doses. The doses were not established a priori but were based on the receptor occupancy results from previous cohort subjects. We started with a pridopidine dose of 90 mg because the exposure at this dose is equivalent to 45 mg twice-daily, which is the most clinically relevant dose currently tested in HD and ALS trials. Because we observed near to complete S1R occupancy with 90 mg, we reduced the next dose to approximately one fourth. This resulted in a dose scheme of 90 (*n* = 3), 22.5 (*n* = 3), 5 (*n* = 2), and 1 mg (*n* = 1). There are two exceptions, 45 mg (*n* = 1) and 0.5 mg (*n* = 1). The investigation with 45 mg showed that a reduction of pridopidine to one half of the dose before would need too many (unnecessary) steps to reach the low dosing range. The lowest dose 0.5 mg (and not 0.25 mg) was chosen to be sure that there is still enough receptor occupancy to be clearly measured with PET. The HD patients (*n* = 3) of the [^18^F] fluspidine PET substudy received 90 mg pridopidine.

In the [^18^F] fallypride substudy, HVs (*n* = 4) received 90 mg pridopidine.

In the test-retest [^18^F] fluspidine PET substudy (HVs, *n* = 2), subjects were investigated at baseline PET1 and PET2 without prior pridopidine treatments to calculate the uncertainty of the RO estimate (Supplementary Materials and Methods).

### Radiochemistry

The injected radioactivity of (S)-(-)-[^18^F] fluspidine ([^18^F]fluspidine) was 279.04 ± 8.16 MBq (mean ± SD). [^18^F] fluspidine was produced as described previously with a modification concerning the final formulation of the tracer [28]. The tracer solution contained 7.5 ml water for injection, 1 ml ethanol, 1.5 ml PEG400, and 100 μl of a concentrated sodium phosphate solution (Braun, Melsungen, Germany). The specific activity was about 180 GBq/μmol at the injection time.

The injected activity of [^18^F] fallypride was 195.38 ± 1.84 MBq. [^18^F] fallypride was prepared according to a published procedure with a specific activity of 600 GBq/μmol at time of injection [29].

### [^18^F] fluspidine and [^18^F] fallypride PET/MR image acquisition and processing

The image acquisition, reconstruction, and processing parameters on the PET/MR system (Biograph mMR, SIEMENS Healthineers, Erlangen, Germany) are described in the Supplementary Materials and Methods in detail [30–32].

### Morphometric MRI analysis

The MRI-scans (T1-MPRAGE) were analyzed morphologically for brain atrophy by calculating the frontal horn width (FH) to intercaudate distance (CC) ratio and the intercaudate distance (CC) distance to inner width (IT) ratio (further detailed in the Supplementary Materials and Methods) [33].

### PET kinetic modeling and data analysis

The total distribution volume *V*_T_ of [^18^F] fluspidine in the brain was computed from the corresponding TACs and the metabolite-corrected arterial input function using a one-tissue compartment model (1TCM) [23]. For the measurement of the reduction of *V*_T_ after oral pridopidine medication, the PET scan was started 2 h after pridopidine administration. To minimize the effect of a changing pridopidine concentration in plasma and tissue during the PET measurement, *V*_T_ was computed from 90 min PET data. Parametric images of the regional distribution volume of [^18^F] fluspidine were created in PMOD (version 3.208, PMOD Technologies, Switzerland) using the Logan plot analysis with t* = 20 min, i.e., the Logan plot becomes linear in all regions after 20 min [34].

The binding potential BP_ND_ of [^18^F] fallypride was computed by the simplified reference tissue model (SRTM) with the cerebellum as reference region [35]. The SRTM was used for the analysis of TAC data and production of parametric images of BP_ND_. Due to the slow kinetic of [^18^F] fallypride binding to the D2/D3R, 210-min PET data were used. Parametric images were created by PMOD.

### Target engagement/receptor occupancy

The receptor occupancy (RO) is defined as the drug treatment-induced reduction of the receptor density. The values of RO are between 0 (no receptor occupancy) and 1 (100% receptor occupancy). RO of pridopidine measured by [^18^F] fluspidine was estimated from the distribution volumes *V*_T_ of all brain regions without (PET1) and post pridopidine treatment (PET2) using Lassen plot analysis. Here, it is assumed that the non-displaceable distribution volume *V*_ND_ and the receptor occupancy RO have the same value in all brain regions [36]. Application of the linear regression

1 $$ {V}_{\mathrm{T}}\left(\mathrm{PET}1\right)-{V}_{\mathrm{T}}\left(\mathrm{PET}2\right)=\mathrm{RO}\left(\ {V}_{\mathrm{T}}\left(\mathrm{PET}1\right)-{V}_{\mathrm{ND}}\right) $$

yielded *V*_ND_ and RO. From the Lassen plot follows

2 $$ \mathrm{RO}=\left(1-\frac{V_{\mathrm{T}}\left(\mathrm{PET}2\right)-{V}_{\mathrm{ND}}}{V_{\mathrm{T}}\left(\mathrm{PET}1\right)-{V}_{\mathrm{ND}}}\right) $$

In case of [^18^F] fallypride, BP_ND_ of a brain region was the outcome parameter of the kinetic modeling. Here, the receptor occupancy was determined from the slope (1-RO) of a modified Lassen plot

3 $$ {\mathrm{BP}}_{\mathrm{ND}}\left(\mathrm{PET}2\right)=\left(1-\mathrm{RO}\right)\ {\mathrm{BP}}_{\mathrm{ND}}\left(\mathrm{PET}1\right) $$

The RO was expressed as:

4 $$ \mathrm{RO}=\left(1-\frac{{\mathrm{BP}}_{\mathrm{ND}}\left(\mathrm{PET}2\right)}{{\mathrm{BP}}_{\mathrm{ND}}\left(\mathrm{PET}1\right)}\right) $$

where BP_ND_ (PET2) is the non-displaceable binding potential after treatment with pridopidine and BP_ND_ (PET1) is the binding potential at baseline.

### Pharmacokinetic parameters of pridopidine

In the case of the S1R ([^18^F]fluspidine) and D2/D3R ([^18^F]fallypride) occupancy substudies, blood samples were collected for the assessment of pharmacokinetic parameters of pridopidine before and following oral pridopidine administration at 0, 0.5, 1, 1.5, 2, 2.5, 3, 3.5, 4, 5, 6, 8, 12, and 24 h, starting 2 h prior PET2. The following pharmacokinetic parameters in plasma were calculated using non-compartmental methods: the average plasma concentration 2 to 3.5 h following drug application (*C*_avg2-4h_), the maximum observed concentration (*C*_max_), the time to reach maximum/peak concentration (*t*_max_), the terminal elimination half-life (*t*_1/2_), and the area under the drug concentration x time curve from time 0 to 24 h (AUC_0-24h_). Furthermore, 4-[3-(methylsulfonyl)phenyl] piperidine (TV-45065), the non-active, main metabolite of pridopidine was determined.

### Statistical analysis

#### Sample size and power considerations

This PET study was exploratory in nature; therefore, no formal hypothesis testing was planned. Based on clinical and practical considerations, a sample size of up to approximately 38 subjects (up to 4 subjects per dose level) was considered adequate for this type of study and to reach the study objectives. Adaptive study design was chosen because it allows increasing or reducing the study total sample size or each dose/time cohort as required. Repeated dose finding committee meetings were executed during this study.

#### Dose S1R occupancy function

The S1R occupancy was described as function of pridopidine dose or concentration in plasma (*C*_avg2-4h_) during the [^18^F] fluspidine PET scan as follows: the Hill equation was chosen to describe the dose and concentration dependency of the receptor occupancy [37].

5 $$ \mathrm{RO}={E}_{\mathrm{max}}\times \left(\frac{c^{\mathrm{n}}}{K_{\mathrm{d}}^{\mathrm{n}}+{c}^{\mathrm{n}}}\right) $$

Here, *c* is the pridopidine dose (mg) or the average pridopidine plasma concentration (ng/ml) between 2 and 4 h after pridopidine treatment. *E*_max_ is the maximal possible receptor occupancy and *K*_d_ the dissociation constant of the receptor/ligand complex but also the dose/concentration was 50% of the maximal possible receptor occupancy is achieved (*K*_d_ = EC_50_). Two models with Hill coefficient *n* fixed to 1 or optimized as a third parameter were investigated and characterized by the Akaike information criterion (AIC). Nonlinear parameter estimation was performed with Mathematica12 (Wolfram Research).

#### D2/D3R occupancy

A paired *t* test (two-tailed) was performed in the case of the D2/D3R occupancy study in HVs (significance at *P* < 0.05).

### Safety and tolerability

All over this investigation, safety and tolerability were documented by monitoring adverse events and conducting laboratory tests, ECGs, physical examinations, and vital sign assessments during each study visit.

## Results

### Demographics and clinical characteristics of HVs and HD patients

Demographics are given in Table 1. In HD patients, there was a direct association between the duration of disease and severity of motor symptoms (TMS), dysfunction of functional capacity (TFC), and semiquantitative measures of brain atrophy. The HD patient with the shortest duration of disease (2 years) had the highest functionality score (TFC = 11, disease stage HD1), best motor function (TMS = 29), and low (close to normal) levels of brain atrophy (CC/IT = 0.15; FH/CC = 2.06). The HD patient with the longest duration of disease (7 years) had the lowest functionality score (TFC = 6, disease stage HD3), worst motor function (TMS = 62), and high levels of brain atrophy (CC/IT = 0.23; FH/CC = 1.46).

**Table 1:** Demographics and clinical characteristics of patients with Huntington disease (HD) and healthy volunteers (HVs)

	HD ([^18^F]fluspidine) mean (SD) [range]	HVs ([^18^F]fluspidine) mean (SD) [range]	HVs ([^18^F]fallypride mean (SD) [range]
*N*	3	11	4
Age (years)	43.3 (13.3) [32–58]	27.2 (1.7) [25–30]	29.0 (4.5) [25–30]
Sex (male/female)	male	male	male
Body weight (kg)	90.9 (35.0) [58.5–128.0]	84.7 (9.2) [63.0–96.1]	88.1 (10.1) [76.3–101.0]
CAG repeat length	45.3 (4.7) [40–49]	n.a.	n.a.
Age-at-disease onset (years)	38.7 (12.5) [30–53]	n.a.	n.a.
Duration of disease (years)	4.6 (2.5) [2–7]	n.a.	n.a.
Baseline UHDRS-TFC	8.7 (2.51) [6–11]	n.a.	n.a.
HD stage	2.0 (1.0) [1–3]	n.a.	n.a.
Baseline UHDRS-TMS	46.3 (16.6) [29–62]	n.a.	n.a.
MRI-atrophy: ratio CC/IT (normal 0.09–0.12)	0.20 (0.05) [0.15–0.23]	0.10 (0.01) [0.08–0.12]	n.a
MRI-atrophy: ratio FH/CC (normal ≥ 2.2)	1.63 (0.38) [1.37–2.06]	3.14 (0.42) [2.60–3.90]	n.a.

Demographics of two male HVs (age 27.5 ± 3.5 years; body weight 67.0 ± 11.3 kg) from the test-retest [^18^F] fluspidine PET study are not shown. CC/IT (MRI measure of caudate atrophy): intercaudate distance to inner table of the skull width ratio; CAG: cytosine-adenine-guanine; FH/CC (MRI measure of caudate atrophy): frontal horn width to intercaudate distance ratio

*HD* Huntington disease, *n.a.* not applied, *range* min–max, *SD* standard deviation, *UHDRS-TFC* Unified Huntington Disease Rate Scale-Total Functional Capacity Score, *UHDRS-TMS* UHDRS-Total Motor Score

### Pharmacokinetics

Pharmacokinetics are detailed in the Supplementary Results (Supplementary Table S1 and Fig. S3). HVs were evaluated after single oral administration of pridopidine at doses ranging from 0.5 to 90 mg. Both pridopidine dose and adjusted weight dose correlated with *C*_avg2-4h_ with high significance (*n* = 18; *r* = 0.927; *P* < 0.0001 and *r* = 0.957, *P* < 0.0001, respectively; two-paired Pearson’s correlation test). *C*_avg2-4h_, *C*_max_, and AUC_0-24h_ showed an increase in plasma levels with increasing pridopidine dose (Table 2; Supplementary Table S1). Mean *C*_max_ was 589 ng/ml from all study subjects receiving 90 mg (HVs, *n* = 6; HD, *n* = 3). This exposure is similar to the exposure measured with 45 mg bid pridopidine (90 mg/day) at steady state in the PRIDE-HD trial (618 ng/ml) [6, 18]. Thus, the S1R and D2/D3R occupancies measured in this PET study after a single dose of 90 mg pridopidine are expected to reflect the levels of receptor occupancy at 45 mg bid steady state. Pridopidine is metabolized (N-depropylated) by the cytochrome P450 enzyme (CYP2D6) to one main inactive metabolite 4-[3-(methylsulfonyl)phenyl] piperidine (TV45065). The concentration at steady state of this inactive metabolite in plasma was less than 10% of the unchanged pridopidine concentration 0 to 12 h after oral administration (Supplementary Fig. S3 and Table S1) [38].

**Table 2:** Sigma-1 receptor occupancy (RO) and pridopidine dose, adjusted weight dose or concentration in plasma (C_avg2-4h_) as assessed by [^18^F] Fluspidine PET at baseline and post-drug in healthy volunteers and patients with Huntington disease ([^18^F] fluspidine study)

Subject	Dose pridopidine (mg)	Adjusted weight dose pridopidine (mg/kg)	Concentration pridopidine (ng/ml)	RO (%)	*V*_ND_
HV1	90.0	1.000	518.0	88.8	4.95
HV2	90.0	1.065	400.0	89.1	5.09
HV3	90.0	1.059	493.0	95.7	4.43
HV4	45.0	0.714	274.0	87.2	3.97
HV5	22.5	0.250	59.1	84.1	4.82
HV6	22.5	0.268	102.0	89.2	4.39
HV7	22.5	0.262	90.6	86.7	5.03
HV8	5.0	0.059	10.0	76.8	4.67
HV9	5.0	0.053	23.5	79.2	4.37
HV10	1.0	0.010	2.4	41.8	6.40
HV11	0.5	0.007	1.3	17.6	1.49
HD1	90.0	1.539	543.0	80.4	4.26
HD2	90.0	0.703	325.0	93.2	3.68
HD3	90.0	1.045	383.0	88.5	4.42

*HD* Huntington disease, *HV* healthy volunteer, *RO* receptor occupancy, *V*_*ND*_ non-displaceable distribution volume

### S1R availability (*V*_T_) in HVs and HD patients ([^18^F]fluspidine)

**Fig. 1:**
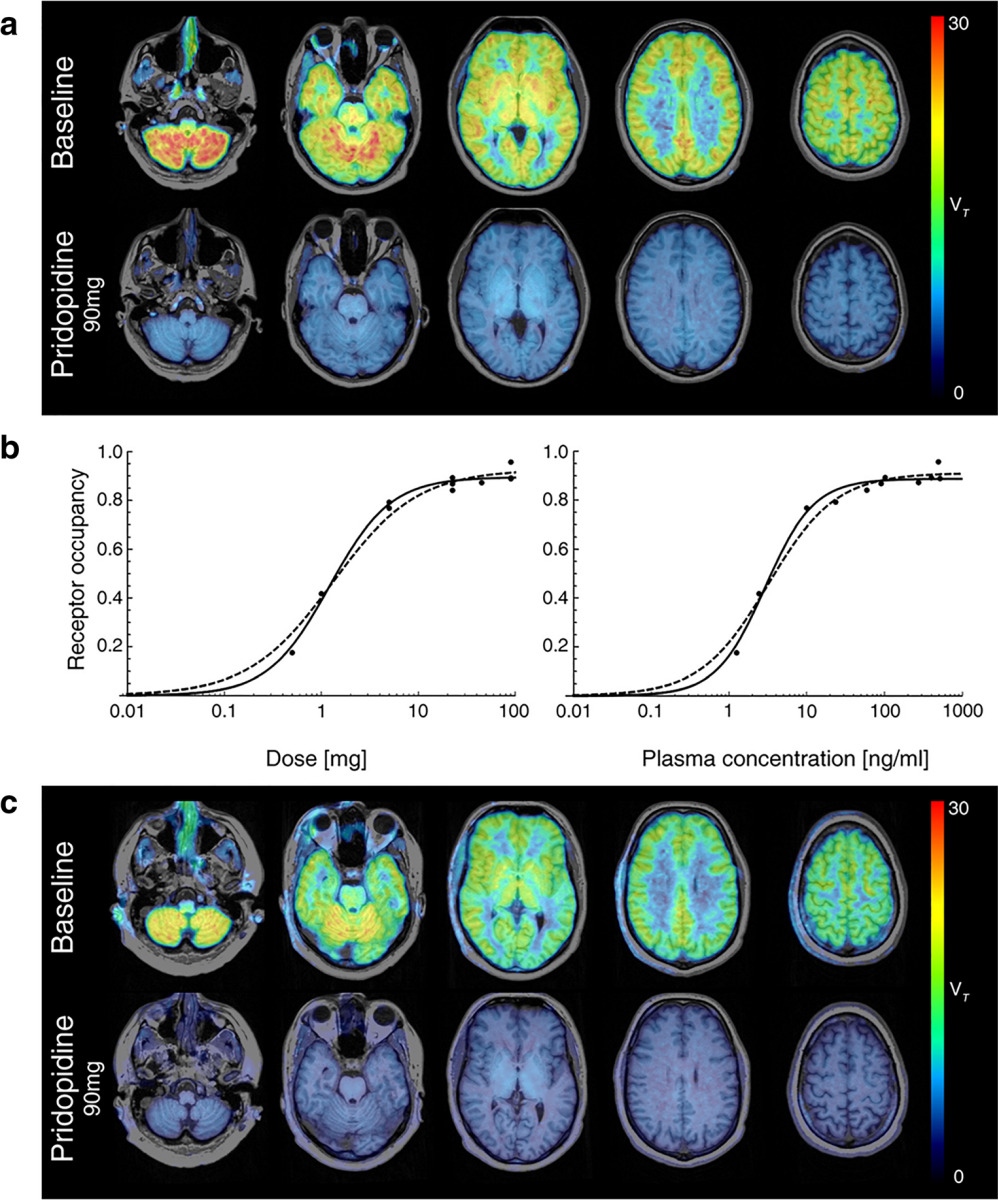
[^18^F] fluspidine baseline and post-drug PET of sigma-1 receptor (S1R) availability in healthy volunteers (HVs) and patients with Huntington disease (HD) and non-linear relationship between pridopidine dose (or plasma concentration) and S1R occupancy. [^18^F] fluspidine PET of S1R availability at baseline and post-drug in HVs and patients with HD. Almost complete S1R engagement/occupancy (*V*_T_) by pridopidine exemplified in one healthy volunteer (**a**) and one HD patient (**c**) is demonstrated within the whole brain at post-drug PET (90 mg pridopidine) as compared with baseline PET. For visualization purpose, parametric PET/MR images are shown. There is a sigmoidal curve relationship (**b**) between the pridopidine dose (left) or plasma concentration (*C*_avg2-4h_; right, both on logarithmic scale) and S1R occupancy in HVs treated by a single dose of pridopidine ranging from 0.5 to 90 mg. The continuous line curve reflects fitting with a three-parameter model (Hill coefficient > 1) which was preferred, whereas the dotted line curve presents a two-parameter type model (Hill coefficient = 1)

In HVs (*n* = 11), exemplified for selected brain regions and representing the physiological S1R availability, mean V_T_ at baseline PET was highest within the cerebellum (26.91 ± 4.49; mean ± SD), moderate within the frontal cortex (21.38 ± 3.37), striatum (19.43 ± 2.95), brain stem (midbrain 19.31 ± 2.70; pons 19.13 ± 3.45; medulla 16.63 ± 2.72), and lowest within the corpus callosum (11.69 ± 2.40). In HD (*n* = 3), mean *V*_T_ at baseline PET was highest within the cerebellum (23.52 ± 12.66), moderate within the frontal cortex (16.32 ± 6.76), striatum (13.59 ± 5.98), brain stem (midbrain 16.46 ± 7.73; pons 16.10 ± 8.74; medulla 14.75 ± 7.28), and lowest within the corpus callosum (8.89 ± 2.75). Compared with HVs, HD patients (*n* = 3) showed lower S1R availability (*V*_T_) in all brain regions (~ 11% to 30%), especially within the striatum. However, group differences in *V*_T_ cannot convincingly be estimated due to the small number of HD patients and large difference of age between HVs and HD patients (Fig. 1; Supplementary Tables S2 and S3).

### S1R occupancy of pridopidine in HVs and HD patients

Individual data of pridopidine dose, weight-adjusted dose, plasma concentration (*C*_avg2-4h_), receptor occupancy (RO), and non-displaceable distribution volume (*V*_ND_) of the HVs and patients with HD are given in Table 2. In HVs, there was a dose-dependent decrease of *V*_T_ throughout the brain following pridopidine administration, as compared with pre-drug PET. Estimation of S1R occupancy by the Lassen plot according to equation (2) is shown for one HV and one HD patient (Supplementary Figs. S4 and S5).

Following application of 90 mg pridopidine, *V*_T_ was highly reduced throughout the brain reaching values of the non-displaceable volume of distribution (*V*_ND_) and representing near to complete S1R occupancy as demonstrated in one HV and one HD patient (Fig. 1; Supplementary Table S2). A dose-response relation for S1Rs occupancy was established in HVs treated with pridopidine at doses ranging from 0.5 to 90 mg. A higher degree of receptor occupancy was observed with higher doses. Doses between 0.5 and 90 mg pridopidine showed S1R occupancy means ranging from 17.6% to 91.2%. Following treatment with 5 mg pridopidine, the RO is 78.0 ± 1.7%. The S1R occupancy reached almost 50% after lowering the pridopidine dose to about 1% of the highest dose of 90 mg. The lowest investigated dose of 0.5 mg pridopidine still caused a RO of 17.6% (Table 2; Supplementary Table S1).

The RO did not differ significantly between HVs and HD after dosing with 90 mg pridopidine, although a larger variance was observed in HD. Administration of 90 mg pridopidine resulted in mean RO of 87.4% (80.4% to 93.2%) in HD patients and of 91.2% (88.8% to 95.7%) in HVs (Fig. 1; Table 2; Supplementary Table S1).

### S1R occupancy as a function of pridopidine dose in HVs

A sigmoidal maximum effect model *E*_max_ was applied to quantify the dose/S1R occupancy relationship [37]. We found a typical Hill curve showing the relation between the pridopidine dose (or plasma concentration) and S1R occupancy in HVs (doses ranging from 0.5 to 90 mg). Regarding this relationship, the two- and three-parameter Hill equations with concentration (ng/ml; Fig. 1b right; Table 3), dose (mg; Fig. 1b left), and adjusted weight dose (mg/kg; Table 3) demonstrated similar results. The Akaike information criterion (AIC) favored the three-parameter model with a Hill coefficient > 1 for all situations (concentration, dose, or adjusted weight dose). The *E*_max_ for both models and situations ranged between 88.7 and 92.6%. EC_50_ (=*K*_d_) and EC_90_, the plasma concentration corresponding to 90% of *E*_max_, for both situation and models were rather low. EC_50_ was similar for both models in the two situations (concentration: 2.90 and 3.17 ng/ml; dose: 1.21 and 1.30 mg). However, EC_90_ (17.57 ng/ml) in the three-parameter model was lower than in the two-parameter model (28.52 ng/ml) for concentration, and similar to that, EC_90_ (7.99 mg) in the three-parameter model was lower than in the two-parameter model (11.73 mg) for dose (Table 3). The EC_90_ value computed with the three- and two-parameter model was 17.57 ng/ml and 28.52 ng/ml, respectively (Table 3), which was about one-tenth or one-fifth of the pridopidine concentration in plasma 12 h after oral application of 45 mg pridopidine (170 ng/ml; Supplementary Fig. S3).

**Table 3:** Pharmacodynamic parameter estimates for two respective models on the relationship between pridopidine dose, weight-adjusted dose, or plasma concentration (*C*_avg2-4h_) and the sigma-1 receptor occupancy in healthy volunteers ([^18^F] fluspidine study)

Model	Parameter	AIC	*E*_max_	EC_50_ (mg or ng/ml)	Hill_coeff._	EC_90_ (mg, mg/kg or ng/ml)
Dose (mg)	3	− 39.48	0.90 (0.01)	1.21 (0.10)	1.38 (0.15)	7.99
	2	− 34.08	0.93 (0.02)	1.30 (0.18)	--	11.73
Weight adjusted dose (mg/kg)	3	− 33.45	0.89 (0.02)	0.014 (0.001)	1.46 (0.22)	0.084
	2	− 29.62	0.93 (0.02)	0.015 (0.002)	--	0.135
Concentration (ng/ml)	3	− 36.01	0.89 (0.02)	2.90 (0.29)	1.39 (0.19)	17.57
	2	− 33.19	0.91 (0.02)	3.17 (0.45)	--	28.52

Numbers in bracket are the standard error

*AIC* Akaike information criterion, *concentration* plasma concentration, *E*_*max*_ maximum effect of the drug, *EC*_*50*_ effective drug exposure associated to 50% of the *E*_max_, *EC*_*90*_ effective drug exposure associated to 90% of the *E*_max_

### Time activity curves of [^18^F] fluspidine PET without and after 90 mg pridopidine

**Fig. 2:**
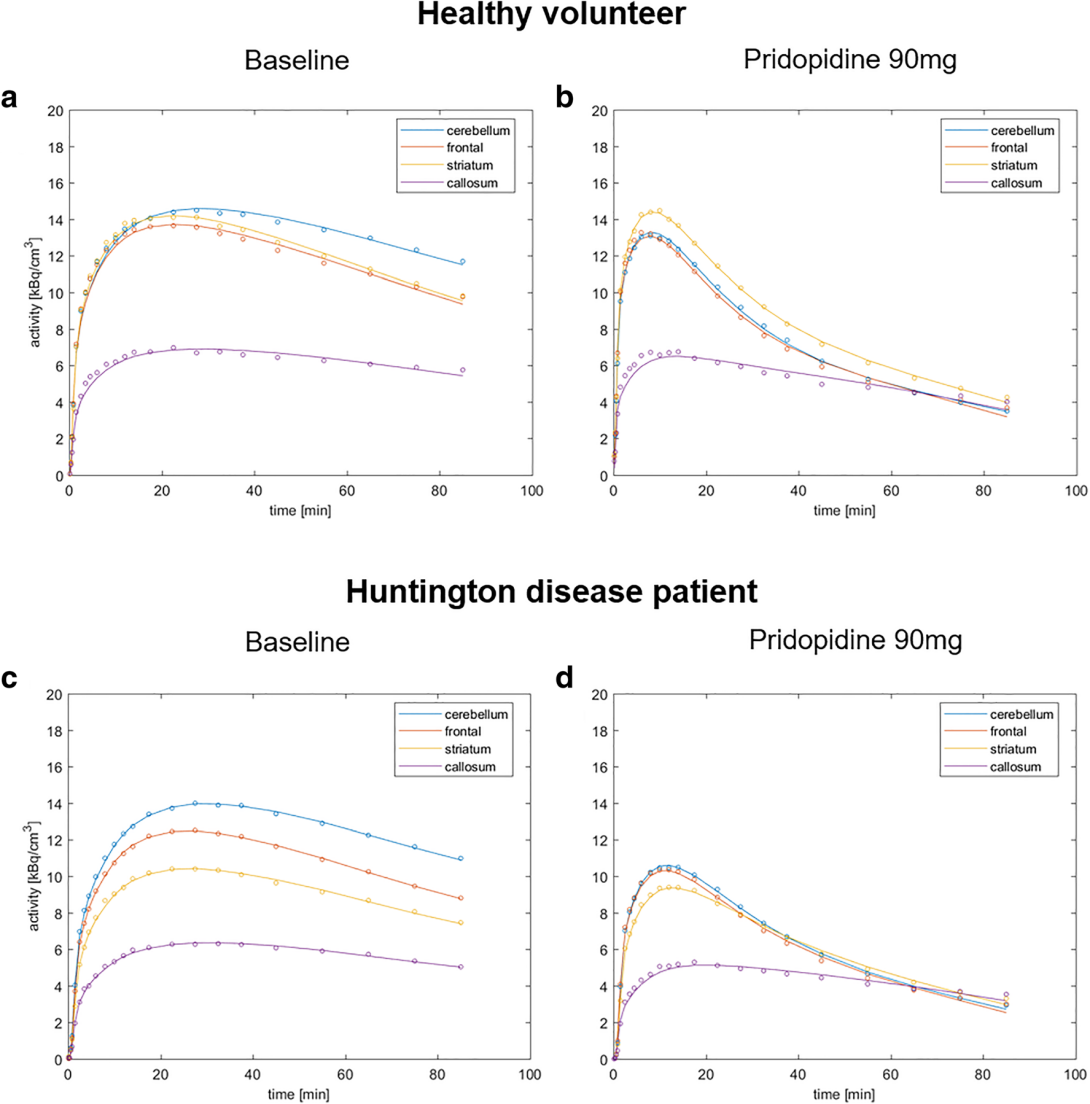
One-tissue compartment model fits of 90 min [^18^F] fluspidine PET data at baseline and post-drug in healthy volunteers (HVs) and patients with Huntington disease (HD). One-tissue compartment model fits of 90 min [^18^F] fluspidine PET data at baseline (**a**, **c**; PET1) and acquired 2 h after oral administration of 90 mg pridopidine (**b**, **d**; PET2) are exemplified for the cerebellum, frontal cortex, striatum, and corpus callosum in one representative HV (**a**, **b**) and one patient with HD (**c**, **d**)

Time activity curves (TACs) of [^18^F] fluspidine in selected S1R-rich regions (cerebellum, frontal cortex, striatum) strongly changed after administration of 90 mg pridopidine as exemplified in one HV (Fig. 2 a and b) and one HD patient (Fig. 2 c and d). Without pridopidine, the TACs in (sub) cortical and cerebellar regions reached a maximum between 20 and 30 min followed by a slow decrease until the end of the PET scan at 90 min (Fig. 2 a and c). With administration of 90 mg pridopidine, due to the large reduction of the distribution volume (*V*_T_), the TACs maximum was already attained between 5 and 10 min after tracer injection followed by a strong reduction in tracer activity until the end of the scan (Fig. 2 b and d). The 1TCM was well suited to describe the tracer dynamics of [^18^F] fluspidine in (sub) cortical and cerebellar regions as could be seen by the very close agreement between measured data points and model predictions by the 1TCM (Fig. 2 a and c).

### D2/D3R occupancy of pridopidine in HVs ([^18^F]fallypride)

**Fig. 3:**
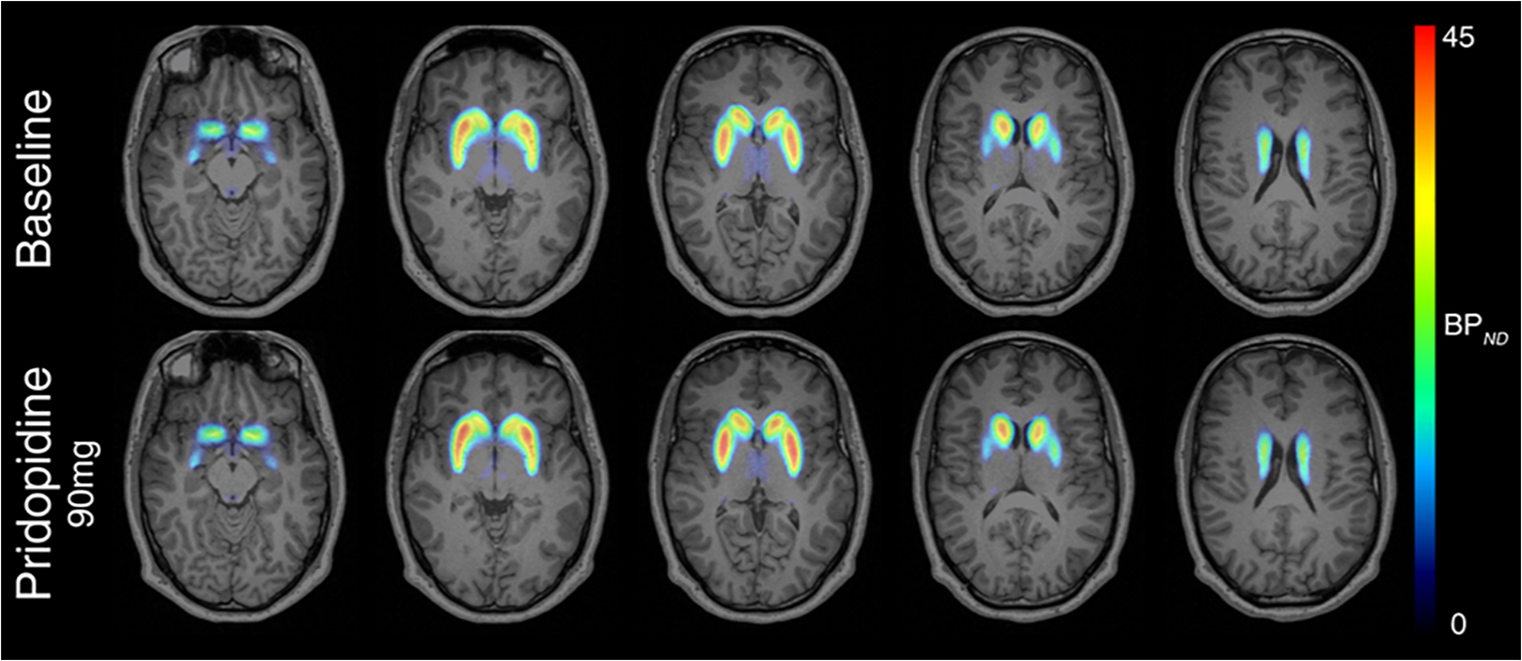
[^18^F] fallypride baseline and post-drug PET of dopamine D2/D3 receptor (D2/D3R) availability in healthy volunteers. Parametric PET/MR images of D2/D3R are demonstrated. There is no D2/D3R occupancy of pridopidine paradigmatically demonstrated in one healthy volunteer as assessed by [^18^F] fallypride PET at baseline and 2 h following single dose of 90 mg pridopidine

The non-displaceable binding potential (BP_ND_) was low for most brain areas except the striatum, which exhibits the highest D2/D3R density. BP_ND_ values within the striatum dropped from 21.44 ± 1.92 before dosing to 20.75 ± 1.99 after administration of 90 mg pridopidine. Analysis of the D2/D3R occupancy in HVs revealed a significant (*P* = 0.047, paired *t* test, two-tailed) but very low RO between 1.8 and 6.1% (mean: 3.3%) after dosing of 90 mg pridopidine (Fig. 3; Table 4; Supplementary Table S4). Estimation of D2/D3R occupancy by the modified Lassen plot according to equation (4) is illustrated in one HV (Supplementary Fig. S6). Due to the minimal D2/D3R occupancy of 90 mg pridopidine in the HVs, it was decided to perform no [^18^F] fallypride PET investigation in the HD patients.

**Table 4:** Dopamine D2/D3 receptor occupancy (RO) and pridopidine dose, weight adjusted dose, and concentration in plasma (*C*_avg2-4h_) as assessed by [^18^F] fallypride PET at baseline and postdrug in healthy volunteers ([^18^F] fallypride study)

Subject	Dose pridopidine (mg)	Adjusted weight dose (mg/kg)	Concentration pridopidine (ng/ml)	RO (%)
HV1	90.0	1.019	507.0	1.8
HV2	90.0	1.180	491.0	3.6
HV3	90.0	1.039	369.0	6.1
HV4	90.0	0.891	226.0	1.8

*HV* healthy volunteer, *RO* receptor occupancy

### TACs of [^18^F] fallypride PET without and following 90 mg pridopidine

**Fig. 4:**
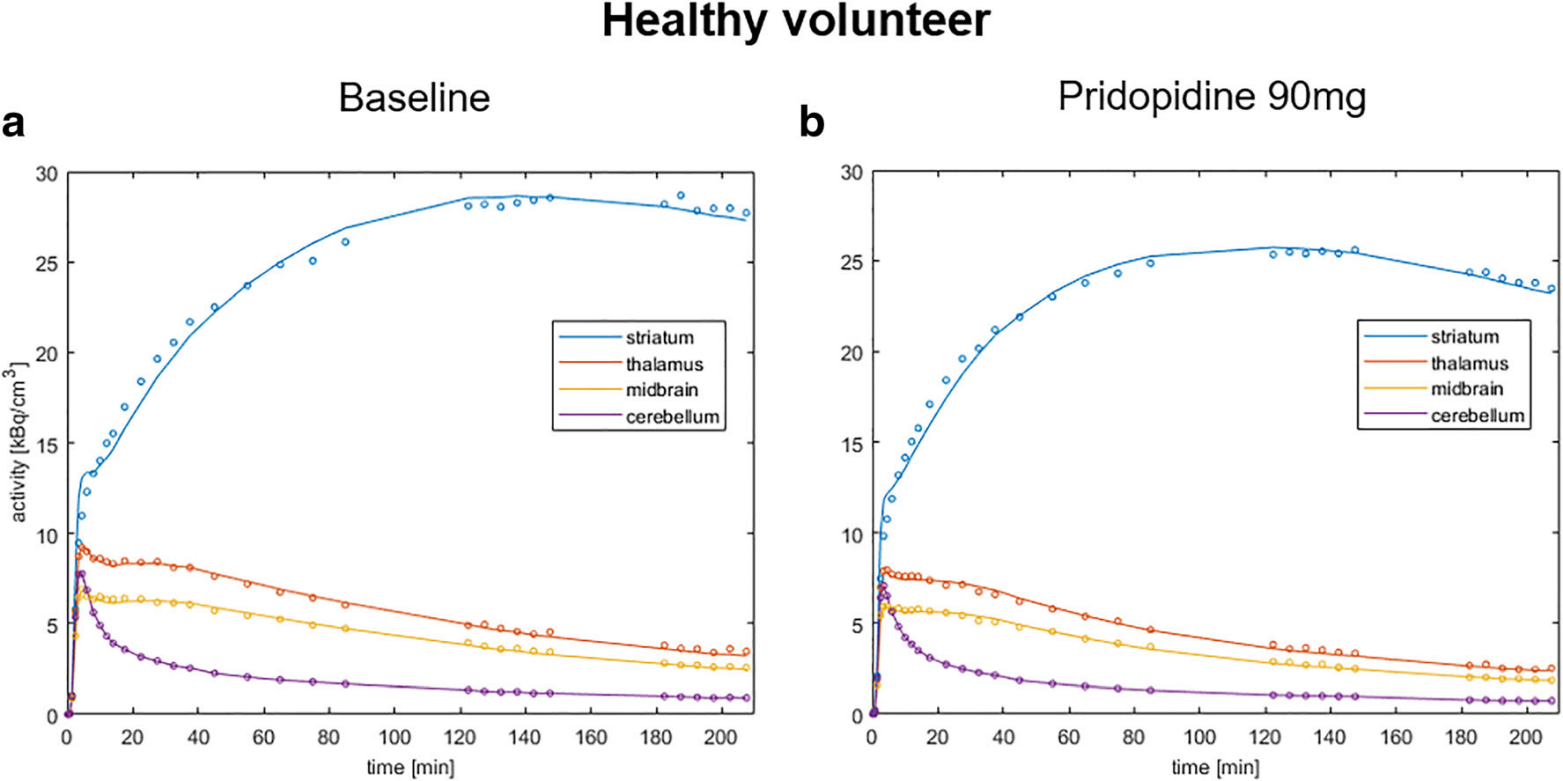
Simplified reference tissue model fits of 210 min [^18^F] fallypride PET data at baseline and post-drug in healthy volunteers (HVs). Simplified reference tissue model fits of 210 min [^18^F] fallypride PET data at baseline (**a**; PET1) and acquired 2 h after oral administration of 90 mg pridopidine (**b**; PET2) are exemplified for the striatum, thalamus, and midbrain using the cerebellum as reference region in one representative healthy volunteer (HV)

The TACs of [^18^F] fallypride did not show any differences before and after pridopidine administration as exemplified in selected brain regions of one HV (Fig. 4 a and b).

### Test-retest study

The test-retest variability of [^18^F] fluspidine PET in two HVs was 0.887 (11.3%; HV#1) and 1.008 (0.8%; HV#2) as estimated by the slope values of the linear regression (Supplementary Fig. S7 and Table S5).

### Blood sampling and metabolite analysis of [^18^F]fluspidine

The fraction of free tracer in plasma, i.e., not bound to plasma proteins, was 0.023 ± 0.007 (*n* = 32) with no difference for subjects studied at baseline and after pridopidine (*n* = 14). Metabolic degradation of [^18^F] fluspidine was faster under pridopidine medication and is positively related to pridopidine dosing in the low-dose range up to 22.5 mg. This effect of pridopidine on [^18^F] fluspidine metabolic degradation is taken into account by the estimation of the distribution volume *V*_T_ by using a metabolite corrected arterial input function analysis (Supplementary Table S6).

### Safety and tolerability

Three HVs did not complete this investigation: one of them due to a methodological problem with the blood data analytics required for the PET investigation; two of them due to adverse events (AEs) of mild intensity (pain after arterial cannulation and abnormal laboratory parameter [hemoglobin]) which were not related to the application of the drug pridopidine or the radioligands. The HVs and patients with HD did not suffer any suspected unexpected serious adverse reactions (SUSARs), serious adverse events (SAEs), or deaths during this study.

## Discussion

Using S1R-selective [^18^F] fluspidine PET, we demonstrate for the first time in vivo a high and selective S1R receptor occupancy (approx. 90%) by pridopidine in HVs and patients with HD, at a dose of 90 mg (plasma exposure correlates to 45 mg bid at steady state). S1R occupancy as a function of pridopidine dose or plasma concentration in HVs can be described by a three-parameter Hill equation with a Hill coefficient larger than 1 for pridopidine doses ranging from 0.5 to 90 mg and respective plasma concentrations. S1R occupancy drops below 50% at a pridopidine dose around 1% of the highest original dose of 90 mg. There are no significant differences in S1Rs occupancies between HVs and patients with HD at 90 mg pridopidine. In contrast, using [^18^F] fallypride PET, we show that the D2/D3R occupancy of pridopidine 90 mg is negligible (~ 3% RO). Resolving pridopidine’s mechanism of action, our PET findings provide significant in vivo evidence for a highly selective and full S1R occupancy in the human brain at a plasma exposure correlating to pridopidine 45 mg bid as previously used in the PRIDE-HD clinical trial (18). Our PET findings in the human brain are in agreement with results of prior preclinical studies. Pridopidine demonstrates in vitro 100-fold and 30-fold higher affinity to the D2R and D3R, respectively [6, 7]. Pridopidine shows in vivo high S1R occupancy vs. low D2/D3R occupancy at behaviorally effective doses in rat brains using [^11^C]SA4503 and [^11^C] Raclopride PET [8].

Neuroprotective properties of pridopidine via S1R-activation have been demonstrated previously in numerous preclinical models of NDD including HD, PD, and ALS [1, 13–17]. These effects of pridopidine are S1R-mediated, because genetic knock-down or pharmacological inhibition of S1Rs abolishes the pridopidine effects [14–16]. Pridopidine shows a S1R-dependent neuroprotective effect against mutant Huntingtin (mHtt)-induced cell death in vitro and in vivo in cortical and striatal neurons in experimental HD mice [39]. Pridopidine decreases motor and behavioral symptoms and rescues transcriptional abnormalities in the striatum via the S1R in a YAC128 mice experimental HD model [39]. Pridopidine enhances BDNF levels in mice brains of experimental HD and PD [13, 15] and upregulates the expression of genes downstream of the BDNF receptor in rat striatum [14]. Pridopidine restores the synaptic activity at neuro-muscular junctions, reduces toxic protein aggregates, ameliorates muscle fiber wasting and enhances BDNF axonal transport in motor neurons carrying the superoxide dismutase 1 (*SOD1*^*G93A*^) mutation [16]. In HD primary neuronal cultures, pridopidine rescues dendritic spine loss and restores the aberrant calcium signaling via the S1R [17]. Taken together, experimental models demonstrate that S1R activation by pridopidine improves motor and psychiatric symptoms and promotes molecular pathways commonly impaired in NDD, such as calcium signaling, mitochondrial function, BDNF expression, integrity of dendritic spines, and transcriptional factors [1, 10, 13, 17, 40]. These S1R-mediated neuroprotective effects of pridopidine previously described in preclinical models of NDD, potentially drive the beneficial therapeutic effects of pridopidine at 45 mg bid observed in patients with HD (PRIDE-HD) [1, 18].

The S1R occupancy is described in this PET study by a sigmoid Hill equation with a Hill coefficient of *n* = 1.38 (dose-dependent), *n* = 1.39 (plasma concentration-dependent), or *n* = 1.46 (adjusted weight dose-dependent). The Akaike information criterion (AIC) always favored the three-parameter model compared with a two-parameter model where n is fixed to 1. The AIC difference between both models was small (< 5) so that the AIC alone did not seem to be sufficient to select convincingly the three-parameter model. Nonetheless, as the crystal structure of the human S1R reveals three potential binding sites, a cooperative ligand binding can be expected [41, 42]. A Hill coefficient larger than 1 is therefore a hint of positive cooperative S1R-pridopidine binding. Thus, using [^18^F] fluspidine PET, we demonstrate for the first time in vivo support for positive cooperative binding of pridopidine to the S1R in the human brain. The *E*_max_ value is well identifiable from the data with a coefficient of variation smaller than 2%. The computed EC_90_ value is found to be relatively low at 8 or 12 mg dose and 18 or 28 ng/ml concentration in plasma for the three- or two-parameter model, respectively. However, the pridopidine concentration in plasma 12 h after oral application of 45 mg pridopidine is 170 ng/ml. This is five-fold higher than the EC_90_ value predicted by the two-parameter model. Therefore, a clinical pridopidine dose of 45 mg bid will guarantee to reach a high S1R occupancy corresponding to the EC_90_ value.

Positive cooperativity binding of pridopidine to the S1R was estimated from the data of the HVs only. But the results have some impact for the HD patients, too. The receptor occupancy curve (equation 5) contains only two parameters *K*_d_ and *n*. *K*_d_ is the dissociation constant of the receptor/ligand complex and the Hill coefficient n can be a measure of cooperative binding. These two parameters depend only on the receptor/ligand system but not on the receptor concentration. As long as the structure of the receptor is not different in two groups, the receptor occupancy curve will be the same in both groups, even if the receptor density in both groups is different. If the S1R system is unmodified in HD patients, the receptor occupancy results from healthy volunteers remain valid. The same holds for the elderly. There may be decrease of receptor density about 5 to 6% per decade in healthy brains. If only receptor density is reduced, the receptor occupancy curve will not change. This PET study was performed in male subjects only. If female subjects had a slightly higher receptor density, this would have no relevance and the receptor occupancy curve estimated from the PET data of healthy male subjects would also be applicable to females. For verification, however, further investigation is required.

Limitations of this study are as follows. As the number of HVs evaluated with 0.5 mg and 1 mg were small (each *n* = 1), our PET findings at these low doses are to be interpreted with caution. The number of subjects that completed the test-retest investigation (*n* = 2) was too low for a detailed statistical characterization. The mean test-retest variability of approximately 6% is in good agreement with other PET studies in the literature. However, further investigation is needed.

Although our estimation of S1R occupancy is based on single-dose data, extensive available pharmacokinetic data at steady state from prior clinical trials with pridopidine enable us to correlate the plasma exposure from our PET study to steady state plasma exposures of known clinical doses. A single oral dose of 90 mg pridopidine results in mean plasma *C*_max_ of 598 ng/ml, which highly correlates with the mean *C*_max_ reached at steady state intake of pridopidine 45 mg bid (618 ng/ml) [6, 18].

Single oral doses of pridopidine ranging from 0.5 to 90 mg were safe and well tolerated by the participants in this investigation. Overall, the safety profile observed in this study was similar to the previously observed safety profile of pridopidine [1, 6, 18]. The mass dose of [^18^F] fluspidine or [^18^F] fallypride used in this study was not sufficient to elicit a pharmacologic response.

## Conclusions

Using PET, we demonstrate for the first time in the living human brain that after a clinically relevant, single oral dose of 90 mg (plasma exposure correlates to 45 mg bid at steady state), pridopidine acts as a selective S1R ligand showing near to complete S1R occupancy (~ 90%) but only minimal (~ 3%) D2/D3R occupancy. The dose S1R occupancy relation suggests positive cooperativity binding of pridopidine to the S1R. Our findings provide clarification about pridopidine’s mechanism of action in the human brain and suggests that previously reported favorable effects of pridopidine 45 mg bid in patients with HD (PRIDE-HD) are mediated via the S1R. Our PET data support further use of the 45 mg bid dose to achieve full and selective targeting of the S1R in future clinical trials of patients with HD and ALS.

## Electronic supplementary material

ESM 1 (PDF 1107 kb)
