# Supplementary material for: Sigma-1 and dopamine D2/D3 receptor occupancy of pridopidine in healthy volunteers and patients with Huntington disease: a [18F] fluspidine and [18F] fallypride PET study
Source: Eur J Nucl Med Mol Imaging. 2020 Sep 29;48(4):1103–15. doi: 10.1007/s00259-020-05030-3 (PMC8041674; doi:10.1007/s00259-020-05030-3)
Supplement: Supplementary file 1 — (PDF 1107 kb) [file 259_2020_5030_MOESM1_ESM.pdf]

Supplementary Material

Sigma-1 and dopamine D2/D3 receptor occupancy of pridopidine in healthy volunteers and patients with Huntington disease: a [^{18}F]Fluspidine and [^{18}F]Fallypride PET study

Igor D. Grachev[1,2]*, Philipp M. Meyer[3]*, Georg A. Becker[3], Marcus Bronzel[4], Doug Marsteller[5], Gina Pastino[5], Ole Voges[4], Laura Rabinovich[5], Helena Knebel[5], Franziska Zientek[3], Michael Rullmann[3], Bernhard Sattler[3], Marianne Patt[3], Thilo Gerhards[3], Maria Strauss[6], Andreas Kluge[4], Peter Brust[7], Juha-Matti Savola[5], Mark F. Gordon[5], Michal Geva[8], Swen Hesse[3], Henryk Barthel[3], Michael Hayden[8], Osama Sabri[3]

***Igor D. Grachev and Philipp M. Meyer contributed equally to this study.**

[1]Teva Branded Pharmaceutical Products R&D, Inc, Malvern, PA 19355, USA; [2]Guide Pharmaceutical Consulting, LLC, Millstone Twp, NJ 08535, USA; [3]Department of Nuclear Medicine; University of Leipzig Medical Center, Leipzig, Germany; [4]ABX-CRO Advanced Pharmaceutical Services Forschungsgesellschaft mbH, Dresden, Germany; [5]Teva Branded Pharmaceutical Products R&D, Inc, Frazer, PA 19355, USA; [6]Department of Psychiatry and Psychotherapy, University of Leipzig Medical Center, Leipzig, Germany; [7]Helmholtz-Zentrum Dresden-Rossendorf, Institute of Radiopharmaceutical Cancer Research, Research Site Leipzig, Leipzig, Germany; [8]Prilenia Therapeutics Development Ltd., Herzliya, Israel.

Supplementary Figures: 7, Supplementary Tables: 6

Clinical Trials.gov Identifier: NCT03019289

EUDRA-CT-Nr. 2016-001757-41

Corresponding author:

Osama Sabri, MD, PhD

Department of Nuclear Medicine

University of Leipzig Medical Center

Liebigstraße 18, 04103 Leipzig, Germany

Osama.Sabri@medizin.uni-leipzig.de

Phone: ++49-341-9718000

Fax: ++49-341-9718129

Supplementary Materials and Methods

Subjects

Twenty male healthy volunteers (HVs) between 25 and 55 years of age, without a clinically significant neuropsychiatric disorder (Diagnostic and Statistical Manual of Mental Disorders, DSM-5), without a history of alcohol, or any other substance dependence in the past 2 years, in good physical and mental health as determined by medical and psychiatric history, suicidality assessment, physical examination, 12-lead ECG, vital signs, clinical laboratory tests, and a MRI scan and without a *CYP2D6* poor metabolizer genotype were enrolled in the study.

Three male patients with Huntington disease (HD) were enrolled who were diagnosed with HD based on clinical features and the presence of ≥36 cytosine-adenine-guanine (CAG) repeats in the huntingtin gene. Patients were at least 25 years of age (symptom onset >18 years), had a body weight ≥50 kg, and a sum of ≥25 points on the Unified Huntington Disease Rating Scale-Total Motor Score (UHDRS-TMS) at the screening visit. Patients were without a clinically significant psychiatric disease, such as major depressive disorder or anxiety according to the DSM-5, without suicidal ideation or attempt at any time in the past or as measured by suicide ideation score of ≥3 on the Columbia-Suicide Severity Rating Scale (C-SSRS), without a history of alcohol, or any other substance dependence in the past 2 years, without a *CYP2D6* poor metabolizer genotype, without a prolonged QTcF interval in the ECG (QTcF interval >450 ms), without a clinically significant heart disease or other severe medical illness. This was assessed by medical and psychiatric history, suicidality assessment, physical examination, 12-lead ECG, vital signs, clinical laboratory tests, and an MRI scan. For the patients with HD taking allowed antipsychotic, antidepressant, or other psychotropic medication, the dosing of medication must have been kept constant for at least 6 weeks before the baseline PET and had to be kept constant during the study.

Inclusion and exclusion criteria

Inclusion criteria: healthy subjects

a. Male subjects between 25 and 55 years (inclusive) of age, with a body mass index (BMI) ≥18.0 to ≤30 kg/m^2 and a body weight of at least 50 kg (inclusive).
b. Good physical and mental health as determined by medical history and psychiatric history, suicidality assessment, physical examination, 12-lead ECG, vital signs, and clinical laboratory tests. A subject with a clinical abnormality in the laboratory profile or BP can be included only if the investigator or his designee considers that the abnormality does not introduce an additional risk factor for the subject's health, or interfere with the study objectives.
c. Ability to understand the requirements of the study; are willing to comply with the requirements of the study (eg, imaging procedures, all dietary, exercise, and alcohol restrictions) and provided their written informed consent to participate in the study.
d. Willingness to provide a blood sample for genetic analyses (including *CYP2D6* status, S1R polymorphs, genetic long QT syndrome in patients who had QT prolongation

following study drug administration or any other genetic analyses related to pridopidine response) at the screening visit.

Inclusion criteria: Huntington disease patients

e. Diagnosis of Huntington disease based on clinical features and the presence of ≥36 CAG repeats in the huntingtin gene.
f. Male age ≥25 years, with an onset of Huntington disease after 18 years of age.
g. Body weight ≥50 kg.
h. A sum of ≥25 points on the UHDRS-TMS at the screening visit.
i. Ability and willingness to provide written informed consent prior to any study related procedure being performed at the screening visit. Patients with a legal guardian should be consented according to local requirements.
j. Willingness to provide a blood sample for genetic analyses (including CAG analysis, *CYP2D6* status, S1R polymorphs, genetic long QT syndrome in patients who had QT prolongation following study drug administration or any other genetic analyses related to pridopidine response or Huntington disease) at the screening visit.
k. Willingness and ability to take oral medication and able to comply with the study specific procedures.
l. Ability to travel to the study center for the duration of the study.
m. Availability and willingness of a caregiver, informant or family member to accompany the patient to the clinic at study visits. The suitability of the caregiver should be judged by the investigator.
n. For patients taking allowed antipsychotic, antidepressant or other psychotropic medication, the dosing of medication must have been kept constant for at least 6 weeks before baseline (visit 2, day -1) and must be kept constant during the study.
o. Ability to understand the requirements of the study; willingness to comply with the requirements of the study (eg, imaging procedures, all restrictions) and provided their written informed consent to participate in the study.

Exclusion criteria: healthy subjects

p. *CYP2D6* poor metabolizers
q. Previous exposure to ionizing radiation or radioactive substances as a result of clinical research or medical treatment in the past 10 years.
r. Large scale tattoos, in particular involving the head and neck area.
s. Counterindication to having an MRI, including (but not limited to):
    - The presence of metal implants (excluding metal dental crowns) that could affect MRI imaging OR
    - has worked with ferrous metals either as a vocation or hobby (for example sheet metal worker, welder or machinist) in such a way that might have led to unknown indwelling of metal fragments that could cause injury if moved in response to the magnetic fields during the MRI imaging.

t. Claustrophobia or needle phobia.
u. Finding on screening MRI that will, in the opinion of the PI impair the safety of the subject or the scientific integrity of the study.
v. Known coagulation abnormality.
w. Parts A and 0 only: Evidence of only one patent arterial supply to the hand (modified Allen test).
x. Current or history of heart condition or increased pro-arrhythmic risk, including:
   - History of cardiovascular disease (eg, coronary artery disease, stroke, arrhythmias, congestive heart failure, uncontrolled hypertension, deep vein thrombosis, pulmonary embolism, family history of thrombophilia)
   - History of Long QT Syndrome or a first degree relative with this condition
   - Family history of sudden death/Brugada syndrome
   - Prolonged Fridericia-corrected QT (QTcF) interval (defined as a QTcF interval of >450 msec) at the screening or admission (baseline) visit(s). If there is evidence of a prolonged QTcF interval from the initial (single) measurement, then the ECG can be repeated twice, and the mean of the 3 screening measurements will be used to determine whether or not the subject is suitable for inclusion in the study.
   - Repolarization deficits
   - Untreated hypokalemia and/or untreated hypomagnesaemia
   - Unclear syncopes
   - Other clinically significant abnormal ECG as judged by the investigator.
y. Creatinine clearance <90 mL/min at screening, calculated using the Cockcroft-Gault equation. It is permitted to repeat the test once, if clinically appropriate.
z. Hemoglobin value below the lower limit of the reference range and evaluated by the investigator to be clinically significant.
aa. Positive serology for HIV-1, HIV-2, HBsAg, and/or hepatitis C.
bb. Presense or history of clinically significant diseases of the renal, hepatic, gastrointestinal, cardiovascular, musculoskeletal, immunological, endocrine (including diabetes even if controlled by diet), metabolic diseases, or other condition known to interfere with the absorption, distribution, metabolism or excretion of drugs, as judged by the investigator.
cc. History of alcohol, narcotic, or any other substance dependence in the past 2 years, as defined by the Diagnostic and Statistical Manual of Mental Disorders, Fifth Edition (DSM-5, American Psychiatric Association 2013).
dd. History of clinically significant psychiatric diseases, such as major depressive disorder or anxiety, as judged by the investigator.
ee. Adverse events of suicidal ideation or attempt at any time in the past or as measured by suicide ideation score of ≥3 on the C-SSRS (screening version), or subjects who answer “Yes” on any of the 5 C-SSRS Suicidal Behavior Items (actual attempt, interrupted attempt, aborted attempt, preparatory acts, or behavior) if the attempt or acts were performed at any time in the past, or subjects who, in the opinion of the investigator, present a risk of suicide.

ff. One of the following conditions:
- major trauma or surgery in the 2 months before screening or at any time between screening and admission
- acute/chronic infection within 2 weeks before screening or at any time between screening and admission
- malignancy within the last 5 years
- epilepsy, seizure, convulsions or syncope (including febrile seizures)
- history of tuberculosis
- BP outside the range of 90 to 139 mmHg (systolic) or 55 to 89 mmHg (diastolic); or pulse rate outside the range of 45 to 99 bpm; all measured after 5 min rest in seated or supine position. Vital signs may be retested twice at intervals of 5 min.

gg. Missing willingness or ability to refrain from intensive physical exercise during the study.

hh. Current or history of clinically significant allergy or known hypersensitivity to any ingredients of the study medication (pridopidine, silicified microcrystalline cellulose, magnesium stearate).

ii. Planned medical treatments (excluding dental care) during the study period, which may interfere with the study.

jj. Use of one of the following prohibited drugs, substances, or foods as follows:
- an investigational drug (new chemical entity) within 6 weeks prior to the first day of study drug administration or within 5 half-lives (whichever is longer)
- any monoamine oxidase inhibitors within 14 d before the first day of study drug administration
- any other medications (including over-the-counter [OTC] medications, vitamins, or herbal or nutritional supplements) within 7 d before the first day of study drug administration (except paracetamol/acetaminophen or ibuprofen used occasionally, up to 24 h before the first day of study drug administration)
- drugs known to significantly inhibit CYP2D6 enzyme drug metabolism within 21 d prior to the first day of study drug administration or within 5 half-lives (whichever is longer) before the first day of study drug administration, or drugs known to significantly induce CYP enzyme drug metabolism within 28 d before the first day of study drug administration
- drugs known to cause significant QT-prolongation such as anti-arrhythmic drugs or antidepressants within 28 d before the first day of study drug administration
- daily consumption of more than 6 units of caffeine and/or xanthine-containing products, within 2 weeks before the first dose of study drug, or not able to refrain from consumption of more than 2 units of caffeine-containing foods or drinks from 48 h prior to admission visit and discharge. One caffeine unit is contained in the following items: 1 cup of coffee, 2 cans of cola, 1 cup of tea, ½ cup of energy drink (eg, Red Bull) or 3 chocolate bars. Subject should be able to abstain from caffeine intake for 20 h during any day.

- cigarette smoking (defined as >5 cigarettes per week) or use of other nicotine-containing products (eg, snuff, nicotine patch, nicotine chewing gum, mock cigarettes, or inhalers). Ex-smokers should have ceased smoking at least 6 months before screening.
- any food or drink/beverage containing alcohol, grapefruit or grapefruit juice, apple or orange juice, vegetables from the mustard green family (eg, kale, broccoli, watercress, collard greens, kohlrabi, brussel sprouts, mustard), and charbroiled meats within 7 d before the first day of study drug administration until after the last day of pharmacokinetic sampling.
- a positive urine drug test or a positive alcohol breath analyzer test at admission visits, or not willing or able to refrain from illicit drugs during the study.

kk. Donation or reception of any blood products (eg, plasma, platelets, etc) in the 1 month before the first day of study drug administration, or has made more than 2 donations within the 12 months preceding the first day of study drug administration, or plans to donate during the study or during the 1 months after the last study visit.

ll. Any of the following reasons:
- The subject is mentally or legally incapacitated, or unable to give consent for any reason.
- The subject is in custody due to an administrative or a legal decision, or under tutelage, or being admitted to a sanitarium or social institution.
- The subject is unable to be contacted in case of emergency.
- The subject is an employee of the site or a relative of an employee at the site.
- Any other reason, at the discretion of the investigator.

Exclusion criteria: Huntington disease patients

a. *CYP2D6* poor metabolizers
b. Previous exposure to ionizing radiation or radioactive substances as a result of clinical research or medical treatment in the past 10 years.
c. Large scale tattoos, in particular involving the head and neck area.
d. Counterindication to having an MRI, including (but not limited to):
- The presence of metal implants (excluding metal dental crowns) that could affect MRI imaging OR
- has worked with ferrous metals either as a vocation or hobby (for example sheet metal worker, welder or machinist) in such a way that might have led to unknown indwelling of metal fragments that could cause injury if moved in response to the magnetic fields during the MRI imaging.

e. Claustrophobia or needle phobia.
f. Finding on screening MRI that will, in the opinion of the PI impair the safety of the subject or the scientific integrity of the study.
g. Severe motor impairment that might cause artifacts.
h. Known coagulation abnormality.

i. Parts A and 0 only: Evidence of only one patent arterial supply to the hand (modified Allen test).

j. Prolonged QTcF interval (defined as a QTcF interval of >450 msec) at the screening visit. If there is evidence of a prolonged QTcF interval at screening from the initial (single) measurement, then the ECG will be repeated twice, and the mean of the 3 screening measurements will be used to determine whether or not the patient is suitable for inclusion in the study.

k. Clinically significant heart disease at the screening visit, defined as follows: (i) significant cardiac event (eg, myocardial infarction), angina pectoris or episode of congestive heart failure with symptoms >Grade 2 New York Heart Association classification within 12 weeks before first admission, or presence of cardiac disease that in the opinion of the investigator increased the risk of ventricular arrhythmia, (ii) history of arrhythmia (multifocal premature ventricular contractions, bigeminy, trigeminy, ventricular tachycardia) that was symptomatic or required treatment (Common Terminology Criteria for Adverse Events Grade 3), symptomatic or uncontrolled atrial fibrillation despite treatment, or asymptomatic sustained ventricular tachycardia, (iii) presence of left bundle branch block, (iv) history of deep vein thrombosis or pulmonary embolism, and/or (v) family history of thrombophilia.

l. Known history of Long QT Syndrome or a first degree relative with this condition.

m. History of epilepsy or of seizures within the last 5 years.

n. Other serious medical illnesses (including but not limited to uncontrolled hypertension, respiratory disease including severe form of asthma, hepatic disease, renal disease, AIDS, unstable psychiatric or other neurologic disorder, endocrine [including controlled diabetes], gastrointestinal, and metabolic diseases) which in the opinion of the investigator may put the patient at risk when participating in the study or may influence the results of the study or affect the patient's ability to take part in the study.

o. Serum potassium, magnesium and/or calcium levels outside of the central laboratory’s reference range at the screening visit and considered clinically significantly abnormal by the investigator. Repeat testing is allowed (up to a maximum of 3 tests) if required to establish whether values are within normal range or clinically significantly abnormal.

p. Medications (within the last 6 weeks prior to baseline [visit 2, day -1]) that have been proven to prolong QT interval or who may require such medications during the course of the study such as, but not limited to, non-allowed anti-psychotic medications, tricyclic antidepressants and/or Class I antiarrhythmics.

q. Medications (within the last 6 weeks prior to baseline [visit 2, day -1]) that are metabolized by CYP2D6 and have the potential of reducing seizure threshold.

r. Creatinine clearance <60 mL/min at screening, calculated using the Cockcroft-Gault equation: (140 - age) × mass (kg) / 72 × serum creatinine (mg/dL). It is allowed to repeat the test once, if clinically appropriate.

s. Any clinically significant, abnormal, screening laboratory result, which in the opinion of the investigator, affects the patients' suitability for the study or puts the patient at risk if he enters the study.

t. Alcohol and/or drug abuse within the 6 months prior to screening, as defined by DSM-5 (American Psychiatric Association 2013).

u. Adverse events of suicidal ideation or attempt at any time in the past or as measured by suicide ideation score of ≥3 on the C-SSRS (screening version), or patients who answer "Yes" on any of the 5 C-SSRS Suicidal Behavior Items (actual attempt, interrupted attempt, aborted attempt, preparatory acts, or behavior) if the attempt or acts were performed at any time in the past, or patients who, in the opinion of the investigator, present a risk of suicide.

v. Known intracranial neoplasms, vascular malformations, history of cerebrovascular accident, or intracranial hemorrhage.

w. Current or history of clinically significant allergy or known hypersensitivity to any ingredients of the study medication (pridopidine, silicified microcrystalline cellulose, magnesium stearate).

x. Treatment with tetrabenazine within 6 weeks of study baseline (visit 2, day -1).

y. Treatment with any investigational product within 6 weeks of screening prior to the first day of study drug administration or within 5 half-lives (whichever is longer) or patients planning to participate in another clinical study assessing any investigational product during the study.

z. Use of one of the following prohibited substances, or foods as follows:

- any food or drink/beverage containing alcohol, grapefruit or grapefruit juice, apple or orange juice, vegetables from the mustard green family (eg, kale, broccoli, watercress, collard greens, kohlrabi, brussel sprouts, mustard), and charbroiled meats within 7 d before the first day of study drug administration until after the last day of pharmacokinetic sampling.
- a positive urine drug test or positive alcohol breath analyzer test at admission visits, or not willing or able to refrain from illicit drugs during the study.

aa. Positive serology for HIV-1, HIV-2, HBsAg, and/or hepatitis C.

Supplementary Materials and Methods (continued)

[^{18}F]Fluspidine or [^{18}F]Fallypride PET/MR image acquisition and processing

Following intravenous application of 279.04±8.16 MBq (mean±SD) [^{18}F]Fluspidine or 195.38±1.84 MBq [^{18}F]Fallypride, dynamic PET scans were acquired on a PET/MR system (Biograph mMR, SIEMENS Healthineers, Erlangen, Germany) from 0-90, 120-150 and 180-210 min in list mode. List mode data from 0-90 min were reconstructed to 23 time frames, the other two scans to 6 frames each giving a total of 35 frames. Attenuation correction of PET data was carried out based on ultrashort echo time (UTE) MR sequences as implemented in the vendors software (VB20P).
For spatial normalization and ROI application, MRI T1-MPRAGE data was acquired (repetition time 1900 ms, echo time 2.53 ms, inversion time 900 ms, slice thickness 1 mm with no gap, image matrix 512 x 512 voxels, in-plane resolution 0.5 x 0.5mm). The PET scans were co-registered to the individual MR image. After spatial normalization of the individual MR to the MNI space using SPM (Wellcome Trust Centre for Neuroimaging, University College London, GB), the same transformation was applied to the PET data. Time activity curves (TACs) for kinetic analysis were generated in 14 brain regions bilaterally (frontal, parietal, temporal, occipital cortices, amygdala, cingulate cortices, hippocampus, cerebellum, striatum, thalamus, corpus callosum, midbrain, pons, medulla, superior longitudinal fasciculus) by applying a region set based on automated anatomic labeling (AAL) and Talairach Daemon (TD) lobes brain atlases to the PET images (Supplementary Fig. S2)[S1,S2].
During the dynamic PET acquisition, arterial radioactivity concentration of [^{18}F]Fluspidine in plasma was measured in up to 38 samples taken from the radial artery. Eleven to 15 blood samples (2 ml) were acquired in the first 3 min after tracer injection followed by 23 samples between 3 and 210 min. One ml plasma was obtained by centrifugation and its activity concentration was measured using a Wizard gamma counter. All activity measurements were decay-corrected to the start time of the PET/MR acquisition. Tracer binding to plasma proteins was determined by ultracentrifugation as described previously. The amount of non-metabolized [^{18}F]Fluspidine was determined in 7 additional arterial blood samples (10 ml) taken at 3, 10, 20, 50, 90, 150, and 210 min post injection by high performance liquid chromatography separation of the parent compound fraction (f_{PC}) followed by gamma counting. Protein-free plasma was obtained from plasma (centrifugation at 4000 U, 5 min) by centrifugation (10 000 U, 10 min) after addition of acetonitrile (2/3; V/V). The fraction of non-metabolized [^{18}F]Fluspidine f_{PC} was fitted by a sum of two exponential functions and used to compute the arterial input function for kinetic modeling [S3].

MRI morphometric analysis

MRI scans (T1-MPRAGE), acquired at baseline PET, were evaluated morphologically by a specialist in radiology and semiquantitatively analyzed for brain atrophy of the caudate head in HVs and patients with HD. Non-HD-pattern or other relevant parenchymal defects were excluded. A progressive atrophy of the caudate head in the patient with HD is the most striking radiological feature and will result in a decrease of the frontal horn width (FH) to intercaudate distance (CC) ratio (abnormal FH/CC ratio < 2.2) or an increase of the intercaudate distance to inner width ratio (threshold for pathologic CC/IT ratio > 0.12)[S4].

Test-retest PET study

The test-retest variability of [^{18}F]Fluspidine binding (V_T) was estimated for two HVs (age 27.5±3.5 yrs; body weight 67.0±11.3 kg) from PET data acquired at the same time point of the day within 11 and 14 weeks. No pridopidine was administered before imaging. The test-retest variability was calculated using linear regression of the first and second PET scan. Test-retest analysis was performed to calculate the uncertainty of the RO estimate. As estimated by the slope values of the linear regression, the variability was 0.887 (11.3%; subject #1) and 1.008 (0.8%; subject #2)(Supplementary Fig. S7; Supplementary Table S5).

Supplementary Results

Pharmacokinetic parameters

$C_{avg2\text{-}4h}$, Cmax and $AUC_{0\text{-}24h}$ increased with increasing dose of pridopidine in the HVs (Supplementary Table S1). Following 90 mg pridopidine, $C_{avg2\text{-}4h}$ was similar in the HVs (470 ng/ml), patients with HD (417 ng/ml) as studied with [^{18}F]Fluspidine, and the HVs as investigated with [^{18}F]Fallypride PET (398 ng/ml). This was the case also for the $AUC_{0\text{-}24h}$ after 90 mg pridopidine for the HVs and patients with HD as studied with [^{18}F]Fluspidine (5709 ng*h/ml and 5391 ng*h/ml, respectively), and the HVs as assessed with [^{18}F]Fallypride PET (5080 ng*h/ml), whereas Cmax showed more variability. For all HVs and patients with HD treated with 90 mg pridopidine (n=10), mean Cmax was 589 ng/ml and mean $AUC_{0\text{-}24h}$ was 5393 ng*h/ml. Cmax was similar as compared to published results from prior clinical trials in patients with HD using 45 mg pridopidine bidaily (Cmax: 618 ng/ml)[S5,S6,S7,S8]. Median t_{max} of pridopidine across all study subjects was between approximately 1 and 2 h. Geometric mean $t_{1/2}$ ranged between 5.3 and 11.6 h for pridopidine over all dose levels in all study subjects, and appeared not to be dose-dependent in the HVs as assessed by [^{18}F]Fluspidine PET (Supplementary Fig. S3; Supplementary Table S1).

Supplementary References

S1. Lancaster JL, Woldorff MG, Parsons LM, Liotti M, Freitas CS, Rainey L, et al. Automated Talairach atlas labels for functional brain mapping. Hum Brain Mapp. 2000;10:120-31.

S2. Tzourio-Mazoyer N, Landeau B, Papathanassiou D, Crivello F, Etard O, Delcroix N, et al. Automated anatomical labeling of activations in SPM using a macroscopic anatomical parcellation of the MNI MRI single-subject brain. Neuroimage. 2002;15:273-89.

S3. Patt M, Becker GA, Grossmann U, Habermann B, Schildan A, Wilke S, et al. Evaluation of metabolism, plasma protein binding and other biological parameters after administration of (-)-[(18)F]Flubatine in humans. Nucl Med Biol. 2014;41:489-94.

S4. Stober T, Wussow W, Schimrigk K. Bicaudate diameter - the most specific and simple CT parameter in the diagnosis of Huntington's disease. Neuroradiology. 1984;26:25-8.

S5. Reilmann R, McGarry A, Grachev ID, Savola JM, Borowsky B, Eyal E, et al. European Huntington's disease network; Huntington study group investigators. Safety and efficacy of pridopidine in patients with Huntington's disease (PRIDE-HD): a phase 2, randomised, placebo-controlled, multicentre, dose-ranging study. Lancet Neurol. 2019;18:165-76.

S6. Johnston TH, Geva M, Steiner L, Orbach A, Papapetropoulos S, Savola JM, et al. Pridopidine, a clinic-ready compound, reduces 3,4-dihydroxyphenylalanine-induced dyskinesia in Parkinsonian macaques. Mov Disord. 2019;34:708-16.

S7. de Yebenes JG, Landwehrmeyer B, Squitieri F, Reilmann R, Rosser A, Barker RA, et al., and the MermaiHD study investigators. Pridopidine for the treatment of motor function in patients with Huntington's disease (MermaiHD): a phase 3, randomised, double-blind, placebo-controlled trial. Lancet Neurol. 2011;10:1049-57.

S8. Huntington Study Group HART Investigators. A randomized, double-blind, placebo-controlled trial of pridopidine in Huntington's disease. Mov Disord. 2013;28:1407-15.

Supplementary Figures and Tables

Supplementary Fig. S1. CONSORT 2010 flow diagram of the trial

CONSORT
TRANSPARENT REPORTING of TRIALS

CONSORT 2010 Flow Diagram

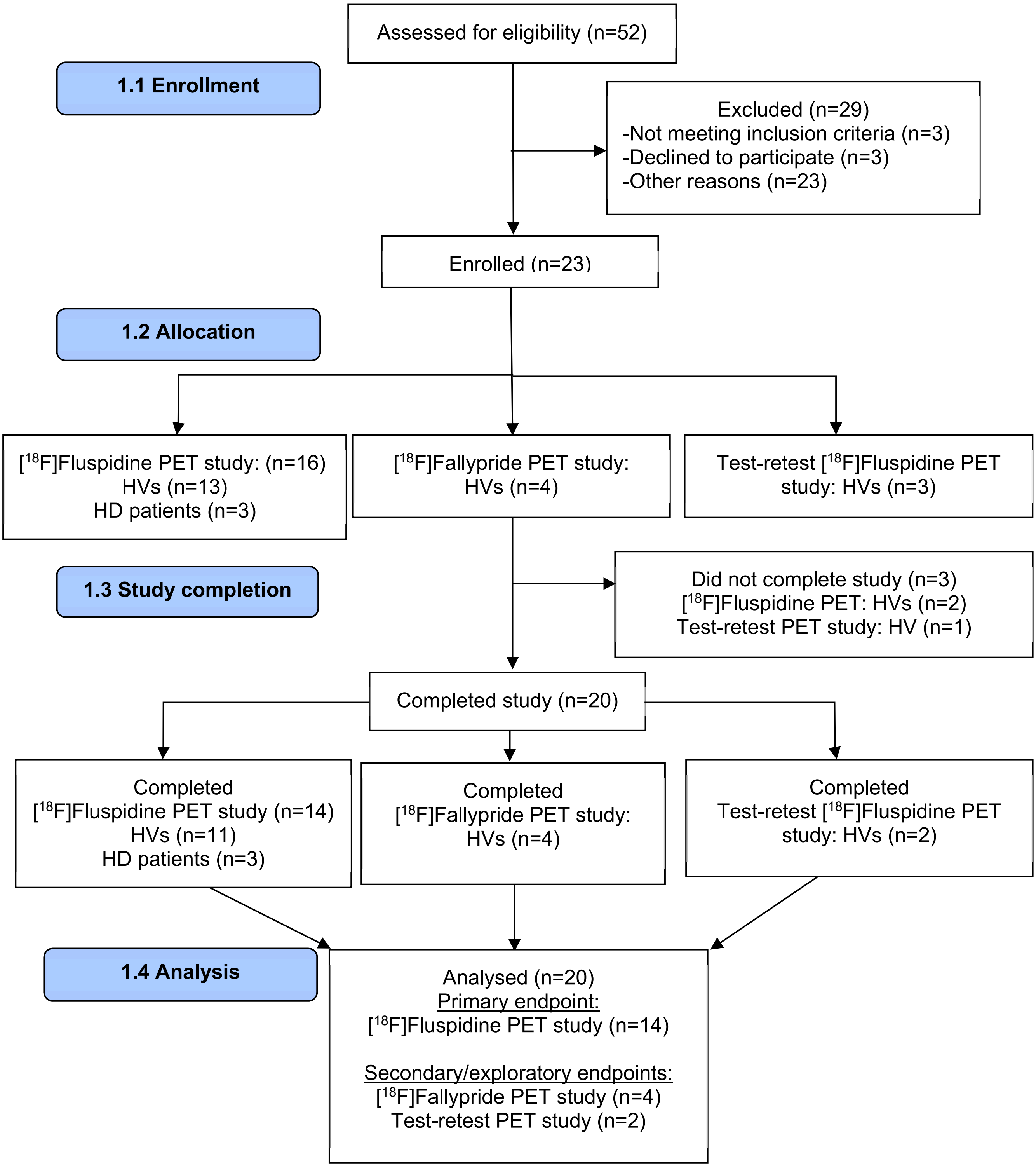

Supplementary Fig. S2. Brain regions analyzed

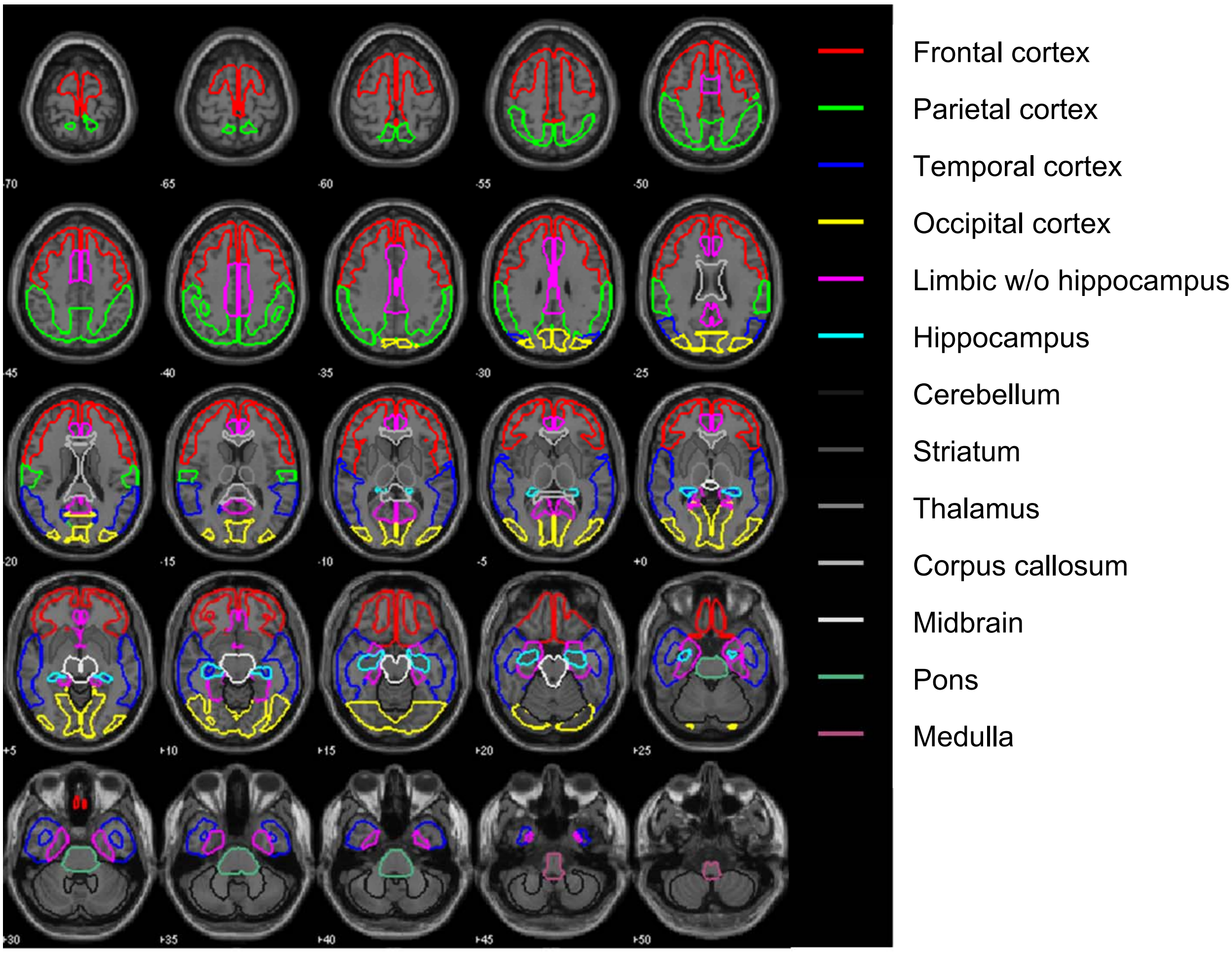

Supplementary Fig. S3: Pharmacokinetics: pridopidine (and non-active main metabolite TV-45065) plasma concentration-time profiles (geometric mean; 0-24 h) for exemplified, single dose cohorts of pridopidine in the healthy volunteers (45 mg and 90 mg) and patients with Huntington disease (90 mg; [^{18}F]Fluspidine PET study).

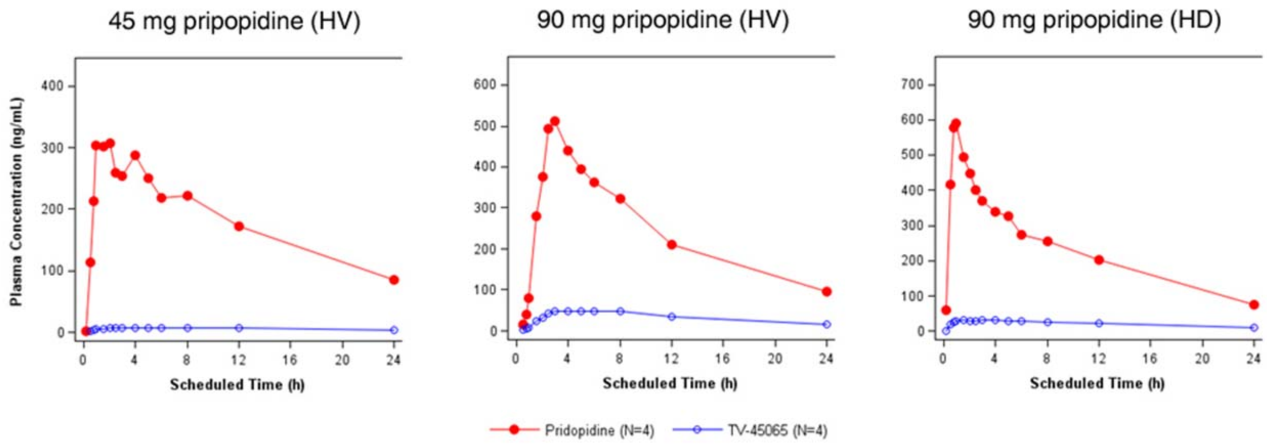

Supplementary Fig. S4. Estimation of the sigma-1 receptor occupancy after oral administration of 90 mg pridopidine by the Lassen-Plot method exemplified in one healthy volunteer. Distribution volume values (V_T) without (PET1) and post pridopidine administration (PET2) were determined from 90 min [^{18}F]Fluspidine PET data.

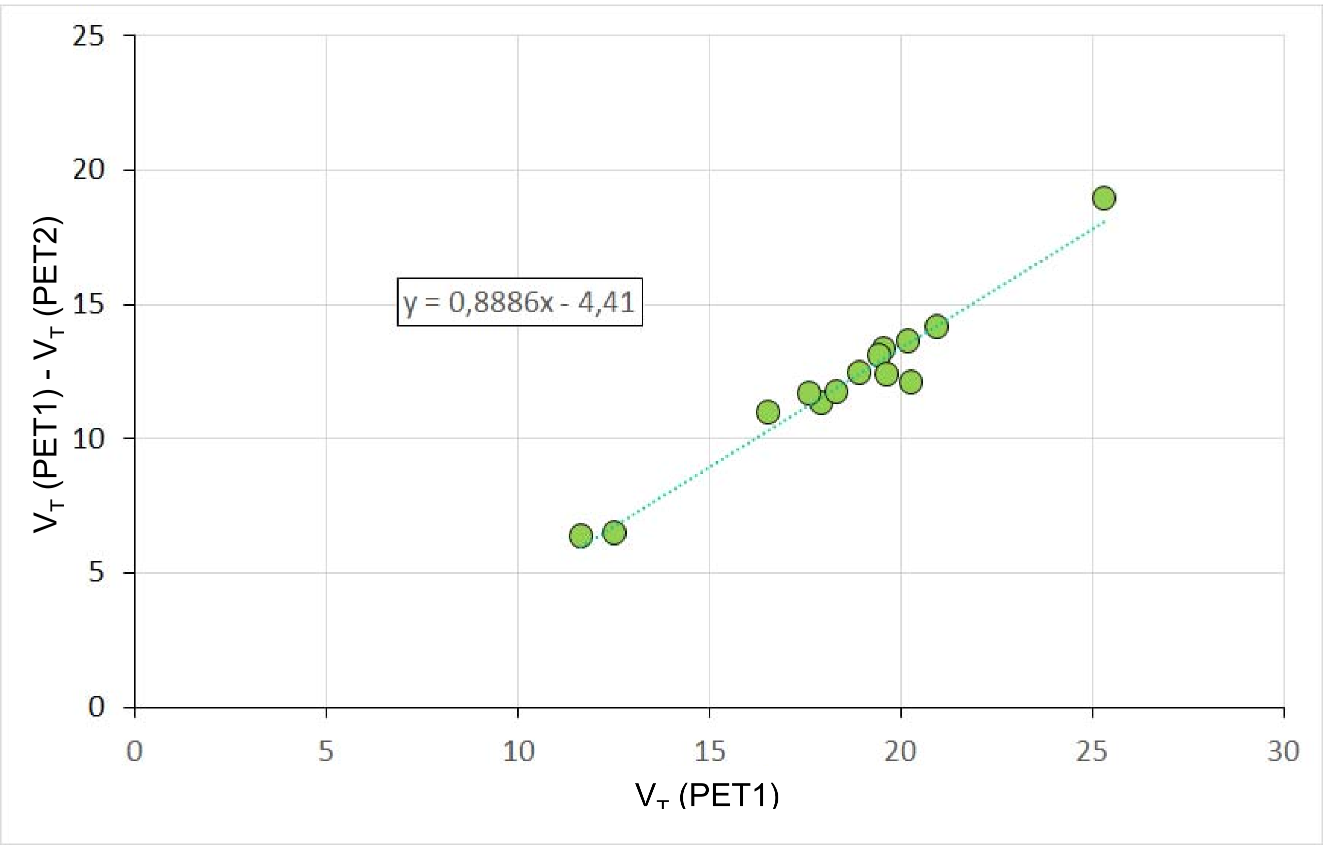

Supplementary Fig. S5. Estimation of the sigma-1 receptor occupancy after oral administration of 90 mg pridopidine by the Lassen-Plot method in one patient with Huntington disease. Distribution volume values (V_T) without (PET1) and post pridopidine administration (PET2) were assessed from 90 min [^{18}F]Fluspidine PET data.

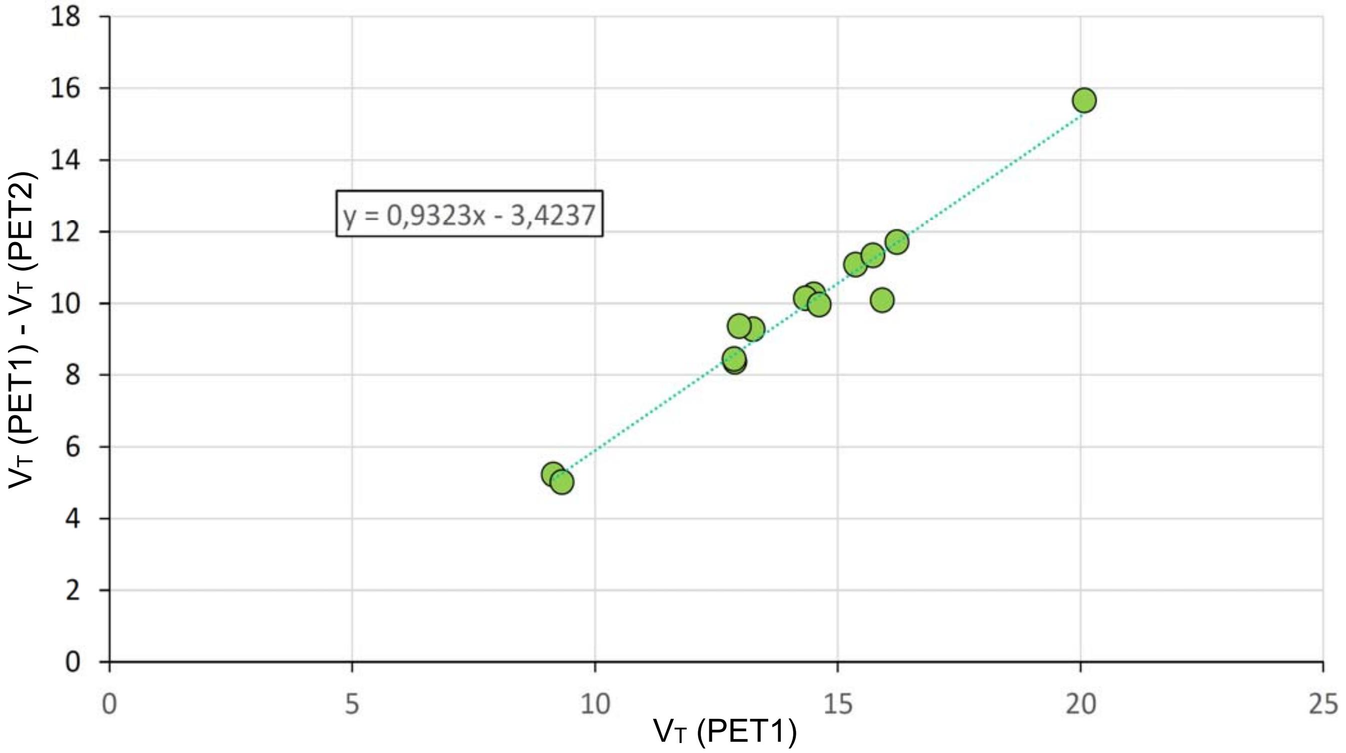

Supplementary Fig. S6. Estimation of the D2/D3 receptor occupancy after oral administration of 90 mg pridopidine by a modified Lassen-Plot method in one representative healthy volunteer. Binding potential values (BP_{ND}) without (PET1) and post pridopidine administration (PET2) were calculated from 210 min [^{18}F]Fallypride PET data.

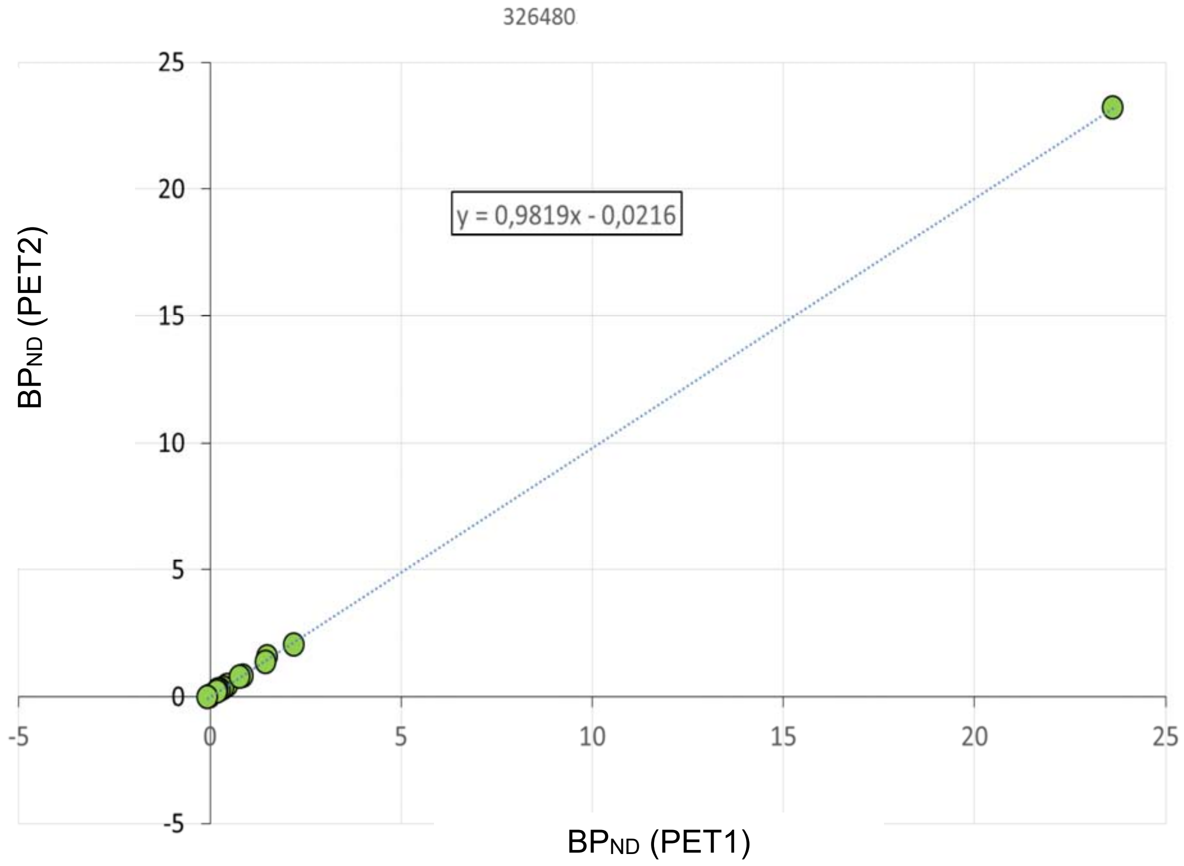

Supplementary Fig. S7. Test-retest variability as determined by linear regression analysis of the distribution volumes (V_T) of the first (PET1) and the second [^{18}F]Fluspidine PET scan (PET2) in two healthy subjects.

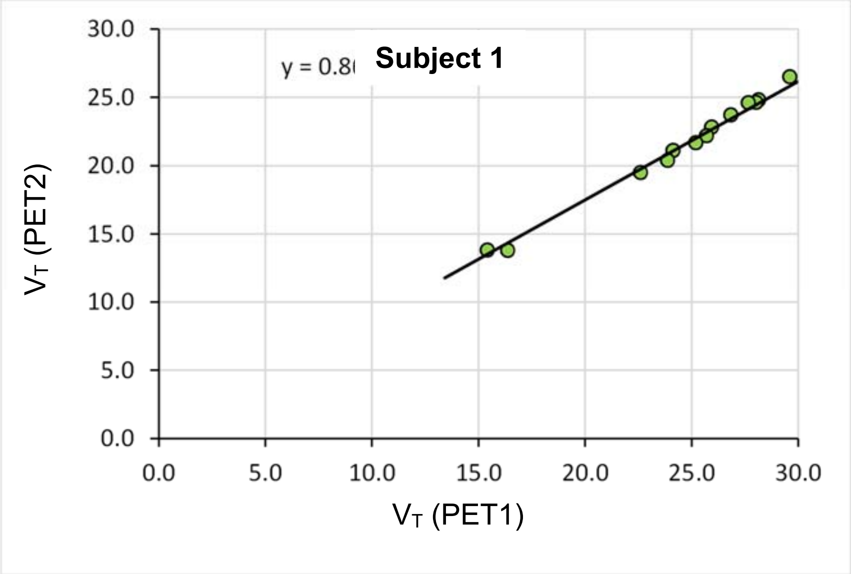

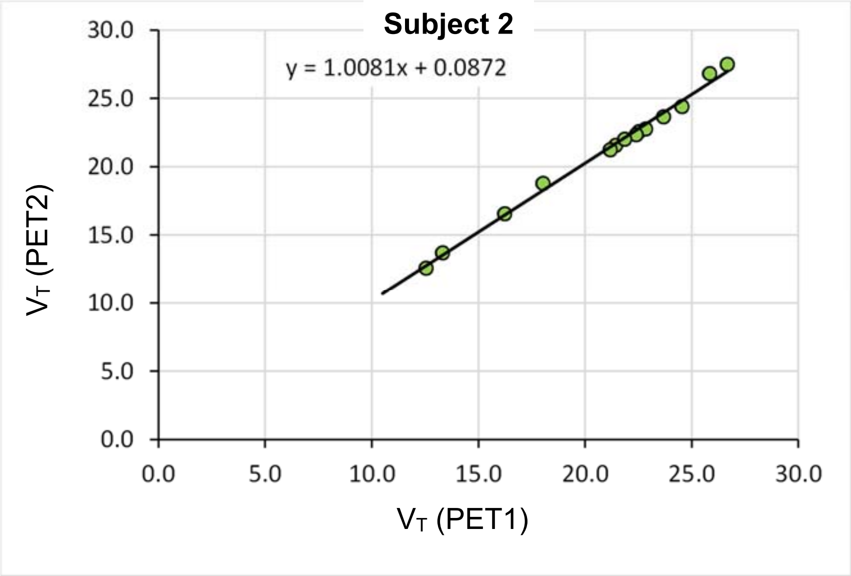

Supplementary Table S1. Pharmacokinetic parameters and their association to pridopidine dose and receptor occupancy (RO).

Substudy	Receptor	Status	Number (n)	Pridopidine (mg)	$C_{avg2-4h}$ (pridopidine) (ng/ml)	$C_{avg2-4h}$ (TV-45065) (ng/ml)	Cmax (ng/mL)	t_{max} (h)	AUC_{0-24h} (ng*h/mL)	$t_{1/2}$ (h)	RO (%)
1	S1R	HV	1	0.5	1.25	0.43	1.33	1.97	12.4	10.1	17.55
1	S1R	HV	1	1.0	2.43	1.47	2.90	1.98	20.6	8.60	41.77
1	S1R	HV	2	5.0	16.7 (9.6)	7.91	25.9 (62.7)	1.00	157 (89.6)	7.50 (82.9)	77.96 (1.71)
1	S1R	HV	3	22.5	83.9 (22.2)	27.9 (4.3)	147 (22.4)	1.00	700 (32.3)	5.27 (18.5)	86.67 (2.59)
1	S1R	HV	1	45.0	274.00	6.7	307	1.98	4177	11.6	87.19
1	S1R	HV	3	90.0	470.3 (62.2)	42.0 (2.3)	552 (10.7)	1.97	5709 (20.2)	9.55 (34.9)	91.21 (3.87)
1	S1R	HD	3	90.0	417 (112.0)	38.9 (26.3)	764 (13.8)	1.00	5391 (61.2)	9.29 (26.9)	87.37 (6.51)
2	D2/D3R	HV	4	90.0	398.3 (130.3)	45.9 (35.4)	452 (42.2)	2.26	5080 (44.2)	8.39 (37.9)	3.34 (2.05)

AUC_{0-24h}: area under the drug concentration x time curve from time 0 to 24h; COV: coefficient of variance; D2/D3R: dopamine D2/D3 receptor; HD: Huntington disease; HV: healthy volunteer; RO: receptor occupancy; S1R: sigma-1 receptor. Numbers are mean if not otherwise given. Numbers in brackets are standard deviation (SD) in the case of $C_{avg2-4h\ (pridopidine)}$, $C_{avg2-4h\ (TV-45065)}$ and RO, median in the case of t_{max}, and COV in % in the case of Cmax, AUC_{0-24h} and $t_{1/2}$. Geometric mean is given for Cmax, AUC_{0-24h} and $t_{1/2}$.

Supplementary Table S2. [18F]Fluspidine PET in healthy volunteers at baseline (PET1) and post-drug (PET2) following pridopidine doses ranging from 0.5 mg to 90 mg p.o.

Pridopidine (mg)	Scan Day	Number Subjects	Activity (MBq)	Start (h p.d.)	V_T in brain regions						
					**Frontal cortex**	**Parietal cortex**	**Temporal cortex**	**Occipital cortex**	**Limbic**	**Hippo-campus**	**Cerebellum**
0.0	PET1	11	275.2±10.7	n.a.	21.38±3.37	20.77±3.49	21.46±3.46	20.19±3.32	22.66±3.64	18.53±2.72	26.91±4.49
90.0	PET2	3	273.3±6.6	2.06±0.10	5.86±0.80	5.79±0.81	6.03±0.84	5.92±0.78	6.22±0.89	6.09±0.80	5.90±0.81
45.0	PET2	1	288.3	2.00	5.82	5.97	6.36	6.10	6.47	6.45	6.72
22.5	PET2	3	279.2±5.7	2.00±0.00	6.95±1.12	6.84±1.11	7.02±1.14	6.80±1.14	7.36±1.24	6.99±1.01	6.98±1.23
5.0	PET2	2	276.4±3.4	2.00±0.00	8.11±2.02	8.02±2.17	8.35±2.19	8.03±2.33	8.72±2.33	8.17±2.09	8.97±2.80
1.0	PET2	1	279.0	2,00	15.10	15.23	15.86	15.53	16.48	14.22	19.02
0.5	PET2	1	282.7	2.00	20.21	19.37	18.83	18.22	20.84	16.90	23.25
					**Striatum**	**Thalamus**	**Corpus callosum**	**Midbrain**	**Pons**	**Medulla**	**SLF**
0.0	PET1	11	275.22±10.7	n.a.	19.43±2.95	21.07±3.12	11.69±2.40	19.31±2.70	19.13±3.45	16.63±2.72	13.02±2.62
90.0	PET2	3	273.3±6.6	2.06±0.10	6.38±0.91	7.41±1.12	4.92±0.40	6.22±0.78	5.58±0.81	4.94±0.97	5.75±0.57
45.0	PET2	1	288.3	2.00	6.90	7.82	4.53	6.29	5.76	4.90	5.63
22.5	PET2	3	279.2±5.7	2.00±0.00	7.23±0.93	8.53±1.43	5.20±0.52	7.09±1.09	6.48±1.14	5.53±0.90	6.15±0.84
5.0	PET2	2	276.4±3.4	2.00±0.00	8.54±2.26	9.67±2.52	6.03±1.42	8.00±2.02	7.35±1.54	6.75±1.21	6.71±1.42
1.0	PET2	1	279.0	2.00	13.68	15.38	8.02	14.37	13.52	11.63	10.89
0.5	PET2	1	282.7	2.00	17.18	19.29	12.76	17.28	19.10	17.13	13.25

h p.d.: hours post-dose; MBq: megabecquerel; n.a.: not applicable; SLF: superior longitudinal fasciculus (white matter); V_T: distribution volume (mL x cm^{-3}).

Supplementary Table S3. [^{18}F]Fluspidine PET in patients with Huntington disease at baseline (PET1) and post-drug (PET2; 90 mg pridopidine).

Pridopidine (mg)	Scan Day	Number Subjects	Activity (MBq)	Start (h p.d.)	V_T in brain regions						
					**Frontal cortex**	**Parietal cortex**	**Temporal cortex**	**Occipital cortex**	**Limbic**	**Hippo-campus**	**Cerebellum**
0.0	PET1	3	286.1±2.9	n.a.	16.32±6.76	15.44±6.19	17.78±8.09	16.25±7.26	17.76±7.58	14.33±6.05	23.52±12.66
90.0	PET2	3	286.6±1.6	2.50±0.87	5.10±0.84	5.03±0.73	5.61±1.23	5.29±1.09	5.62±1.07	5.58±1.08	5.84±1.62
					**Striatum**	**Thalamus**	**Corpus callosum**	**Midbrain**	**Pons**	**Medulla**	**SLF**
0.0	PET1	3	286.1±2.9	n.a.	13.59±5.98	17.35±7.48	8.89±2.75	16.46±7.73	16.10±8.74	14.75±7.28	11.63±5.07
90.0	PET2	3	286.6±1.6	2.50±0.87	5.29±1.04	6.91±1.45	4.20±0.27	5.87±1.44	5.36±1.63	4.76±1.26	5.54±1.15

h p.d.: hours post-dose; MBq: megabecquerel; n.a.: not applicable; SLF: superior longitudinal fasciculus (white matter); V_T: distribution volume (mL x cm^{-3}).

Supplementary Table S4. [18F]Fallypride PET in healthy volunteers. Binding potential (BP_{ND}; mean±SD) for baseline (PET1) and post-drug PET (PET2) in the healthy volunteers following administration of 90 mg pridopidine.

Pridopidine (mg)	Scan Day	Number Subjects	Activity (MBq)	Start (h p.d.)	BP_{ND} in brain regions						
					**Frontal cortex**	**Parietal cortex**	**Temporal cortex**	**Occipital cortex**	**Limbic**	**Hippo campus**	**Cerebellum(*)**
0.0	PET1	4	196.1±2.0	n.a.	0.40±0.10	0.29±0.15	0.77±0.19	0.23±0.10	0.71±0.11	1.42±0.10	0.00±0.00
90.0	PET2	4	194.7±1.9	2.00±0.00	0.41±0.07	0.30±012	0.73±0.16	0.24±0.09	0.70±0.09	1.42±0.13	0.00±0.00
					**Striatum**	**Thalamus**	**Corpus callosum**	**Midbrain**	**Pons**	**Medulla**	**SLF**
0.0	PET1	4	196.1±2.0	n.a.	21.44±1.92	2.13±0.16	0.09±0.17	1.45±0.06	-0.04±0.04	0.27±0.05	-0.10±0.04
90.0	PET2	4	194.7±1.9	2.00±0.00	20.75±1.99	1.99±0.12	0.12±0.16	1.37±0.03	-0.02±0.02	0.25±0.04	-0.04±0.02

*Cerebellum as reference region; BP_{ND}: binding potential; h p.d.: hours post-dose; MBq: megabecquerel; n.a.: not applicable; SLF: superior longitudinal fasciculus (white matter).

Supplementary Table S5: Test-retest study. [^{18}F]Fluspidine PET in healthy volunteers at two time points (PET1 and PET2) to analyze the normal variability of sigma-1 receptor availability as assessed by the distribution volume (V_T) within the brain.

Subject ID	Scan Day	Activity (MBq)	V_T in brain regions						
			Frontal cortex	Parietal cortex	Temporal cortex	Occipital cortex	Limbic	Hippo-campus	Cerebellum
1	PET1	278.0	28.11	26.82	27.99	25.92	29.59	24.13	35.09
1	PET2	278.5	24.83	23.72	24.65	22.83	26.52	21.13	29.67
2	PET1	279.9	23.67	22.51	22.83	21.40	24.54	21.19	26.68
2	PET2	275.7	23.66	22.59	22.78	21.56	24.41	21.24	27.50
**Subject ID**	**Scan Day**	**Activity (MBq)**	**Striatum**	**Thalamus**	**Corpus callosum**	**Midbrain**	**Pons**	**Medulla**	**SLF**
1	PET1	278.0	25.68	27.63	15.42	25.19	23.84	22.59	16.39
1	PET2	278.5	22.20	24.63	13.85	21.69	20.39	19.52	13.83
2	PET1	279.9	22.39	25.84	12.51	21.82	18.03	16.22	13.32
2	PET2	275.7	22.35	26.82	12.58	22.02	18.78	16.57	13.71

MBq: megabecquerel; SLF: superior longitudinal fasciculus (white matter); V_T: distribution volume (mL x cm^{-3}).

Supplementary Table S6. Comparison between baseline PET and post-drug PET: the fraction of non-metabolized radioligand [^{18}F]Fluspidine (parent compound) as exemplified at 3, 50, 90 and 210 min p.i. Values are given as mean (SD) if applicable.

**Pridopidine dose (mg)**	**Number (n)**	**Time (min p.i.)**			
		**3 min p.i.**	**50 min p.i.**	**90 min p.i.**	**210 min p.i.**
0	18	0.94±0.04	0.58±0.11	0.36±0.08	0.21±0.05
0.5	1	0.99	0.55	0.38	0.22
1	1	1.00	0.50	0.30	0.25
5	2	0.90±0.00	0.38±0.24	0.27±0.15	0.20±0.04
22.5	3	0.96±0.02	0.34±0.05	0.22±0.04	0.20±0.04
45	1	0.93	0.41	0.25	0.24
90	6	0.95±0.04	0.29±0.08	0.20±0.04	0.16±0.04
